# A Probabilistic Palimpsest Model of Visual Short-term Memory

**DOI:** 10.1371/journal.pcbi.1004003

**Published:** 2015-01-22

**Authors:** Loic Matthey, Paul M. Bays, Peter Dayan

**Affiliations:** 1 Gatsby Computational Neuroscience Unit, University College London, London, United Kingdom; 2 Sobell Department of Motor Neuroscience and Movement Disorders, UCL Institute of Neurology, London, United Kingdom; 3 Institute of Cognitive and Brain Sciences, University of California, Berkeley, Berkeley, California, United States of America; University of Tübingen and Max Planck Institute for Biologial Cybernetics, GERMANY

## Abstract

Working memory plays a key role in cognition, and yet its mechanisms remain much debated. Human performance on memory tasks is severely limited; however, the two major classes of theory explaining the limits leave open questions about key issues such as how multiple simultaneously-represented items can be distinguished. We propose a palimpsest model, with the occurrent activity of a single population of neurons coding for several multi-featured items. Using a probabilistic approach to storage and recall, we show how this model can account for many qualitative aspects of existing experimental data. In our account, the underlying nature of a memory item depends entirely on the characteristics of the population representation, and we provide analytical and numerical insights into critical issues such as multiplicity and binding. We consider representations in which information about individual feature values is partially separate from the information about binding that creates single items out of multiple features. An appropriate balance between these two types of information is required to capture fully the different types of error seen in human experimental data. Our model provides the first principled account of misbinding errors. We also suggest a specific set of stimuli designed to elucidate the representations that subjects actually employ.

## Author Summary

Humans can remember several visual items for a few seconds and recall them; however, performance deteriorates surprisingly quickly with the number of items that must be stored. Along with increasingly inaccurate recollection, subjects make association errors, sometimes apparently recalling the wrong item altogether. No current model accounts for these data fully. We discuss a simple model that focuses attention on the population representations that are putatively involved, and thereby on limits to the amount of information that can be stored and recalled. We use theoretical and numerical methods to examine the characteristics and performance of our model.

## Introduction

The ability to store information about the world and use it at a later time is a critical aspect of human cognition, and comes in many different forms. One such is visual short term memory, which holds visual information for brief intervals, for example to make a decision or complete a task. Since it is important in many contexts, it has been the subject of a wealth of psychophysical and neurophysiological investigations, and offers constraints on coding and representation as well as on pure storage.

**Figure 1:**
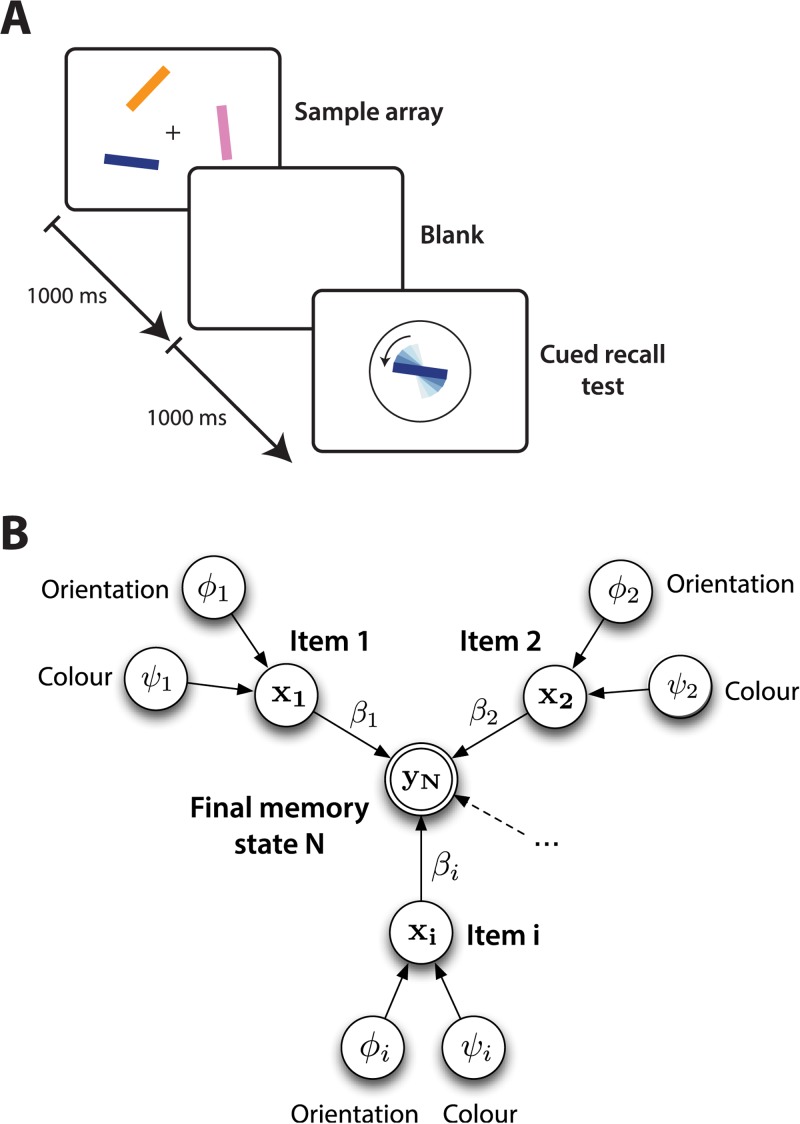
Experimental paradigm and storage process. (**A**) The experimental paradigm. First, an array of items was shown for 1000ms, followed by a 1000ms blank screen. Next, a probe with the colour of one of the items in the array was presented, but at a random orientation. Subjects had to adjust the orientation of the probe item to match that of the relevant item in the original array. (**B**) Graphical model of the storage process. Several items *i*, each composed of two features (here, orientation *ϕ_i_* and colour *ψ_i_*), eliciting individual responses **x_i_** in a neuronal population code, are combined together additively to form a final memory state **y_N_**.

Here, we consider a paradigmatic visual short-term memory experiment from [1] which is illustrated in Fig. 1A. Subjects were presented with an array of oriented coloured bars. After a short presentation time, the array was removed and one of the coloured bars was re-presented at a random orientation. The subjects had to rotate the bar back to its previously presented orientation (the target orientation) from memory. Thus multiple items must be stored, each having two features (colour and orientation). One such feature is cued (here colour), and the associated other feature (orientation) had to be recalled.

As one might expect, the mean precision of recall (typically defined as the inverse of the standard deviation of the errors) decreases with the number of items, and does so smoothly. However, along with small deviations from the target orientation, subjects can sometimes make large errors. This effect has historically been explained by considering that memory can only store a small number of items, in a finite number of “slots” [2–5]. Items not allocated a slot cannot be recalled even approximately, and so are assumed to be guessed (leading to large errors). The number of slots has been estimated to be fairly low for most individuals (∼4 items), although it can be expanded significantly by explicit training [6].

More recently, several groups have proposed alternative mechanisms for storage [1, 7–11] based on the metaphor of a divisible, but limited resource. This resource is allocated amongst all the items that are stored, rather than only some being remembered at all. However, as more items are stored, each receives less of the resource, hence decreasing the precision of storage and/or recall.

**Figure 2:**
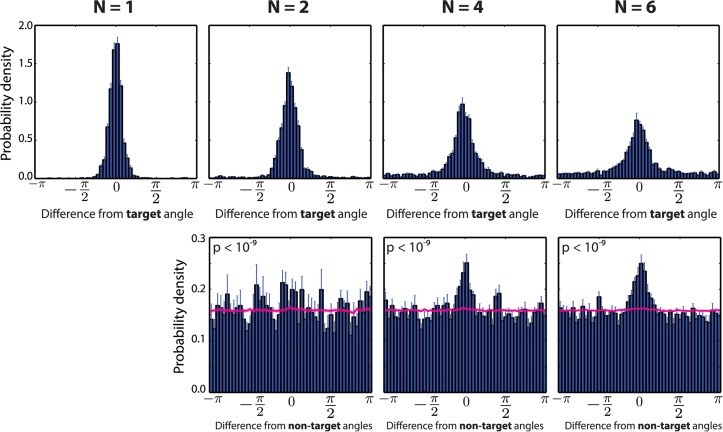
Distribution of errors in human subjects. **Top**: Probability of errors between recalled and target colour (this particular experiment cued the location and required colour to be recalled), for 1, 2, 4 and 6 items (shown simultaneously). One can see that the tail of the distribution grows when an increasing number of items is stored. **Bottom**: Errors relative to non-target values presented in each array. Any bias towards 0 indicates misbinding. Error bars show one standard error of the mean, for 8 subjects. A resampling-based estimation of the probability of misbinding error was performed, and the p-value for a non-zero non-target response component is shown for N = 2, 4 and 6. Misbinding errors are significantly present in all conditions. See Methods for a description of the resampling analysis. The magenta lines (and outline showing standard error of the mean) show histograms obtained from randomly sampling from mixture models derived from the resampling analysis, removing inter-item correlations. Recalculated based on [7].

One key battleground for this debate has been the observation of characteristic, so-called misbinding errors [7, 8, 12]. These arise when subjects recall the orientation of another item with which they are presented (that of a “non-target”) instead of that of the target. Fig. 2, reanalysed from [7] shows this for a task in which colour had to be recalled based on a cued location (items did not have an orientation in this task). On the upper row, the distribution of errors around the correct target colour is shown; each plot is for a different number *N* of items in the array. The responses are distributed around the correct target colour, with a dispersion increasing with *N*. A characteristic baseline error level, increasing with set size, is also visible. This uniform baseline has been interpreted as the signature of guessing [5]. The lower row of graphs shows how this dispersion hides misbinding, by indicating the distributions of deviations between the response and all other non-target items. The presence of a significantly higher proportion of small such deviations is a sign of responses incorrectly reporting other non-target items. We measured the significance using a resampling procedure (see Methods and the Misbinding errors section for details) that ensure that the effect is not just due to the increased probability of being close to an item when their overall number increases; rather, it arises from biases in the recall process.

Finite resource models provide a more natural account of misbinding than classical slot models. This is because all items are stored to some fidelity, making it possible that subjects recall the wrong item in some circumstances [1, 7, 13]. However, a formal theory of these circumstances is presently lacking.

Further, although resource models have been successful in explaining psychophysical data, there is as yet no canonical implementation, or agreement about what exactly is the limited resource. One suggestion is that it is the total number of spikes available in a population of neurons [14, 15], using normalization [15, 16] or by otherwise limiting the number of bits available to store the items [17]. However, accounts based on versions of this solve in a rather unusual way the problem of “multiplicity”, i.e., when multiple items need to be represented simultaneously. That is, they typically employ distinct and separable storage for each possible item (i.e., effectively an unbounded number of slots), with the competition coming from restricting the total amount of activation across all storage units. This leaves unclear the mechanics of allocation of these distinct pools, which is key to misbinding.

Here, we consider a different model in which a single set of storage units is employed, with different items being overlaid, as in a palimpsest [18–21]. A conventional palimpsest is a manuscript which has been partly scraped-off or cleaned before being written upon again, allowing past inscriptions to be recovered along with the most recent content. Similarly, we consider the case where multiple items are written on top of each other in the same neuronal population. For a paradigm in which items are presented sequentially, partial erasure would occur between each presentations. However, for the sake of simplicity, here we only consider paradigms in which all items are presented simultaneously, and so without erasure of the palimpsest in between. We will refer to this as a restricted palimpsest storage process. Depending on the representations used and how patterns decay and combine, the final memory state of the neuronal population will retain a trace of all items that have been written onto it. From this final memory state, we then consider a Bayesian probabilistic recall process starting from the cued feature, mimicking the experimental paradigm presented above.

Recall performance in our model depends sensitively on the representation used to store different items in the memory. We consider two specific examples that we call “mixed” and “hierarchical”. These are intended as paradigm cases of a wider range of possibilities, rather than be fully comprehensive; we analyse their characteristics empirically and theoretically. One particularly important aspect for both codes is the balance between allocating units to storing information about individual feature values, and storing binding information to link each item’s features together. This translates, through the medium of probabilistic recall, into a balance between two of the types of experimentally observed error mentioned above: the small displacements from the target item, and the more theoretically elusive misbinding, here rendered as a (slightly displaced) recall of one of the non-target stored items. The third type of random guessing errors also arise in the model via probabilistic recall, even though all items are actually stored. The relative frequencies of these errors varies with the nature of the population code and the number of items stored.

A classical way to quantify the quality of population codes is the Fisher information (FI). The FI cannot be used to capture the frequency of misbinding—we therefore provide a thorough empirical characterization of the model’s production of this sort of error. However, the FI does correctly determine the width of the distribution of responses around either target or non-target items—the displacements mentioned above.

We show how it may be possible to distinguish between particular population codes based on available experimental data, and so propose new experiments that focus on the interplay between simultaneously-stored stimuli, which could shed light on how items interact in human working memory. Note that the goals of this paper are to introduce and explore population palimpsest memories rather than to fit psychophysical data in quantitative detail.

We start by presenting the three key facets of our model: representation, storage and recall. We consider its empirical and theoretical properties, relative respectively to data from existing visual short-term memory experiments and to the Fisher information, which characterizes memory fidelity. This raises the complex issue of misbinding, which we treat in some detail, for both a classical feature-based representation, and a hierarchical representation that we then describe. Finally, we consider specific arrangements of targets in the space of possible memories that are expected to lead to patterns of errors that can help distinguish between different representations.

## Results

We propose a model of representation, storage and recall in visual working memory. By considering all aspects together, we show how to accommodate a range of experimental findings with a small set of assumptions. To be concrete, we consider the experimental paradigm shown in Fig. 1A (based on [1]), and described above. Here, each item is determined by two features: angle and colour, both of which are taken as being angular (as the latter can be encoded as an angle on a colour wheel).

### Representation

Consider the case of a population of *M* units representing the memory of all items seen in a trial. The simultaneous population activity of these units is read during recall to infer the feature of the item of interest. The finiteness of the population, the nature of the representation employed and the influence of noise jointly constitute the limited resource associated with our memory.

In terms of the representation, we assume that units have continuous firing rates, and are tuned to specific combinations of features. Unit *i* has a preferred angle and colour, with separate tuning widths to each feature, and its mean activity follows a Von Mises curve as shown in Equation (1). We use Bivariate Von Mises [22, 23] tuning curves as they provide a convenient parametrisation of the sensitivity to a pair of angular features.

$$\mu_{m}\left(\phi ,\psi \right)=\frac{1}{4\pi^{2}I_{0}\left(\tau_{1,m}\right)I_{0}\left(\tau_{2,m}\right)}\exp\left(\tau_{1,m}\cos\left(\phi -\theta_{m}\right)+\tau_{2,m}\cos\left(\psi -\gamma_{m}\right)\right),$$ (1)

Here, *ϕ* and *ψ* are respectively the orientation and colour of the item to be represented. *θ*_*m*_ and *γ*_*m*_ are the preferred angle and colour of unit *m*. *τ*_1,*m*_ and *τ*_2,*m*_ are called concentration parameters, which control the size of the receptive field, as well as the sensitivity of each unit to the different features. Units have continuous valued firing-rate responses, and suffer from independent Gaussian noise about these mean activities. To examine the scaling behaviour of the model, we use a normalization scheme that constrains the mean summed overall network activity induced by any item to be constant as the receptive field concentrations change (although the total activity in the memory grows with the number of items stored). We use independent Gaussian noise for simplicity, although it would be straightforward to examine a more neurally plausible, Poisson, noise model.

Writing *x*_*m*_ as the firing rate of unit *m*, the population activity **x** = [*x*_1_,…,*x*_*M*_]^*T*^ is

$$x\;|\;\phi ,\psi \;∼\;N(\mu \left(\phi ,\psi \right),\;\sigma_{x}^{2}I)$$ (2)

**Figure 3:**
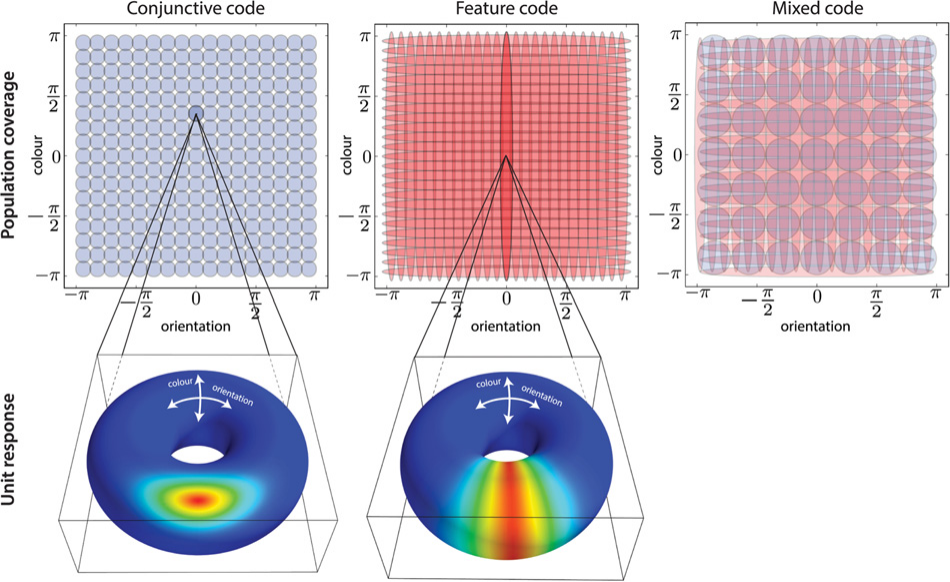
Example population codes. **Top**: Receptive fields of units (one standard deviation), shown for the three different types of population codes: conjunctive, feature and mixed. **Bottom**: Activity profile over the entire stimulus space for the two shaded units on the left.

Depending on the distribution of *τ*_1,*m*_ and *τ*_2,*m*_, several types of population code can be generated (see Fig. 3 and 10). *τ*_1,*m*_ = *τ*_2,*m*_ = *τ* ∀*i* corresponds to a “conjunctive” population code, in which each unit is sensitive to a combination of the two features. Conversely, a “feature” population code employs two subpopulations; one has *τ*_1,*m*_ = *τ*,*τ*_2,*m*_ = 0, and is sensitive only to the first feature; the other has *τ*_1,*m*_ = 0,*τ*_2,*m*_ = *τ*, and is only sensitive to the second. We also consider a “mixed” population code including both conjunctive and feature units, and entertain various possibilities for the relative proportions of the two types. This “mixed” population code provides an easy way to parametrise the relative information required to store feature values accurately (these are mostly encoded in the feature sub-population) versus the binding information required to link features together into item-like representations (only encoded by the conjunctive sub-population). Moreover, the different types of population code will require different number of neurons to cover the entire stimulus space appropriately. This will become increasingly important as the number of features increases.

We study the effects of different types of representation on the nature and quality of recall, and show that aspects of human experimental data are better accounted for by population codes that might at first seem sub-optimal.

### Storage and recall process

According to our restricted palimpsest memory process (“restricted”, because, as mentioned in the introduction, we do not assume erasure of the palimpsest between storage steps), the noisy population activities associated with all the items are simply summed to produce the final memory. As can be expected, the characteristics of the representation used will determine how readily possible it is to extract items when they are overlaid.

The storage process is depicted in Fig. 1B, in which *N* items are stored simultaneously. Again, for simplicity, assuming that the final memory suffers from spherical Gaussian noise, we derive:

$$x_{i}\;|\;\phi_{i},\psi_{i}\;∼\;N\left(\mu \left(\phi_{i},\psi_{i}\right),\sigma_{x}^{2}I\right)$$ (3)

$$y_{N}\;|\;\;x_{1},\;...\;,x_{N}\;∼\;N\left(\overset{N}{\underset{i=1}{\sum }}\beta_{i}x_{i},\;\sigma_{y}^{2}I\right)$$ (4)

**Figure 4:**
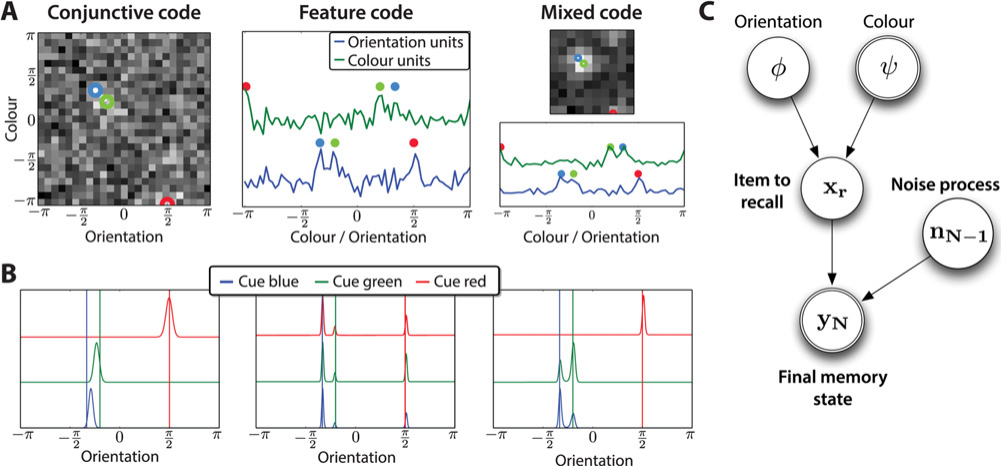
Recall model and posterior for different population codes. (**A**) Example memory states for the different population codes, when three items are stored. Coloured circles indicate the veridical feature values. Left: Conjunctive population code, involving little interaction even between nearby items. Middle: Feature population code. Right: Mixed population code — a few conjunctive units provide just enough binding information to recall the features associated with the appropriate items. (**B**): Cued posterior probabilities, given the veridical colour to be recalled (the three curves correspond to cueing the three possible colours; vertical bars indicate the true stored orientations). (**C**) Graphical model representation of the process of recall. The final memory state and colour are observed; the orientation must be inferred.

Here, *ϕ*_*i*_ and *ψ*_*i*_ represent the feature values of item *i*. **x_i_** is the population representation of item *i*. Multiple items are then summed to produce the final memory state **y_N_**. Extraction of stored information is based on the memory state **y_N_**, along with any prior information. Examples of memory states for a chosen set of stimuli and population codes are shown in Fig. 4. For completeness, these expressions include two generalizations that we do not consider further here: the terms *β*_*i*_ allow different items to be stored with different strengths in the memory (to accommodate tasks involving explicit attentional instructions); however, here we set *β*_*i*_ = 1 ∀*i*. The parameter $\sigma_{y}^{2}$ allows for extra memory noise, but is set to a very small value in our experiments (*σ*_*y*_ = 10^−5^).

Having produced this final memory state, the next step is to recall the correct feature based on the recall cue. Bayes optimal recall would require marginalising over the non-target items that were simultaneously presented. Given the final memory state **y_N_**, and a cued feature value (e.g. a colour *ψ*), this would lead to the posterior distribution over the value of the other feature of this item. However, this marginalisation would be computationally penal, since it would likely require explicitly representing and processing all the non-cued items. Instead, we make the simplifying assumption that only the item to be recalled is explicitly modelled, with the non-targets being collapsed together and treated as background noise. Conceptually, this corresponds to extracting a specific item of interest out of irrelevant noise. This approach was adopted by [21, 24, 25], in the context of retrieval from long-term memory in multistate synapses.

The algorithm is illustrated in Fig. 4. Given a memory state **y_N_** and the cued colour *ψ*, we compute the posterior distribution over *ϕ* explicitly (Fig. 4B). No closed-form solution exists for this posterior in general, because of the non-linear transform associated with the population code ***μ***(⋅). Therefore we sample from it using slice sampling [26]. We treat a single sample as the output of recalling a feature from our model for this trial. The use of sampling instead of a maximum likelihood (or MAP) solution has two main consequences: the variance of the posterior has a direct effect on the variance of the recalled orientation, and multi-modal posteriors will reflect situations in which another orientation may be reported in place of the appropriate one.

We formalize this process by writing **m_N–1_** as the contribution of the noise process to the mean of the final memory state and Σ_**N**_ as the contribution of the noise to the full memory covariance, see Fig. 4C. *r* is the index of the item to be recalled, which we integrate over, as it is unknown during recall.

$$y_{N}\;|\;\phi ,\psi ,r\;∼\;N\left(m_{N-1}\;+\;\beta_{r}\mu \left(\phi ,\psi \right)\;,\;\Sigma_{N}\right)\;$$ (5)

$$\phi \;|\;y_{N},\psi \;\propto \;p\left(\phi \right){\int^{\text{​}}}^{\text{​}}dr\;p\left(r\right)\;p(y_{N}\;|\;\phi ,\psi ,r)$$ (6)

This posterior is usually peaked around the appropriate orientation; however, depending on the population code used and the number of stored memories, additional modes can appear (see Fig. 4B, middle and right). These correspond to the effects of noise and other items on the recall of the item of interest; the latter allows us to study the question of binding.

We now consider various characteristics of our models in the context of visual short-term memory experiments.

### Modelling visual working memory experiments

**Figure 5:**
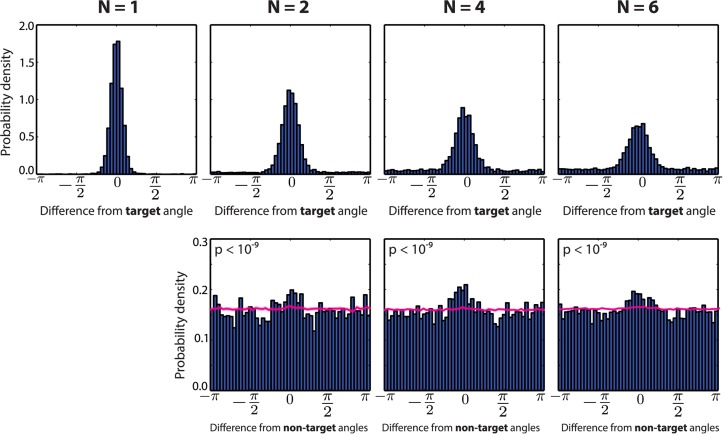
Distribution of errors of the model. The model is capable of recreating error distributions seen in the literature, such as those shown in Fig. 2. (**Top row**) Distribution of errors around the target angle. The central bump is at 0^*o*^, showing that recall is normally accurate. The distribution has a non-zero baseline which combines all sources of error. (**Bottom row**) Distribution of errors relative to all non-target angles. A central tendency in those plots has been interpreted as supporting evidence for the presence of misbinding errors in the responses. Histogram computed on 5000 samples of the model (no standard deviation is shown as all samples are equally probable) The p-values for a resampling analysis of the non-target mixture proportion are shown in each panel. The null hypothesis of no misbinding error can be significantly rejected for all item numbers. The magenta curves represent the resampling-based histograms assuming no misbinding error. Mixed population, *M* = 200, *σ_x_* = 0.25.

First, the model reproduces the baseline, apparently uniform, component of the distribution of errors (see Fig. 5 upper row, compared to Fig. 2). However, this does not arise from pure random guessing. Rather, a sample is always taken from the posterior distribution given a memory state composed after storing all items. Nevertheless, interactions between items and the overall background noise in the memory imply that the model sometimes samples values away from the target, so producing output resembling guessing. On the lower row of Fig. 5, we see that our model can also reproduce misbinding errors, shown by the over-abundance of small errors towards non-target items values during recall. This central tendency is reduced compared to the experimental data from Fig. 2B, but is still significantly present. In addition, the magenta curve and penumbra represent the distribution of samples from the model when inter-items correlations have been removed.

The second experimental observation captured by the model is the decrease in recall precision as a function of the set size, which is the number of stored items. Here we study the precision of recall using the procedure defined by [7]. This involves fitting a mixture of Von Mises components on the recall samples, using a procedure based on the EM algorithm [27]. This mixture model consists of one Von Mises component per item (target or non-target) and a uniform random component. All Von Mises components share a single concentration parameter *κ*. This mixture model approach turns out to be substantially more robust to outliers than computing the circular standard deviation of the raw errors directly. We refer to *κ* as the memory fidelity, and show how it depends on set size. In addition to this memory fidelity, two other types of errors are specifically captured by this analysis: misbinding errors, the probability of recalling from a non-target, and random errors, the probability of recalling from the uniform random component. These will be analysed more thoroughly in the Misbinding errors section.

**Figure 6:**
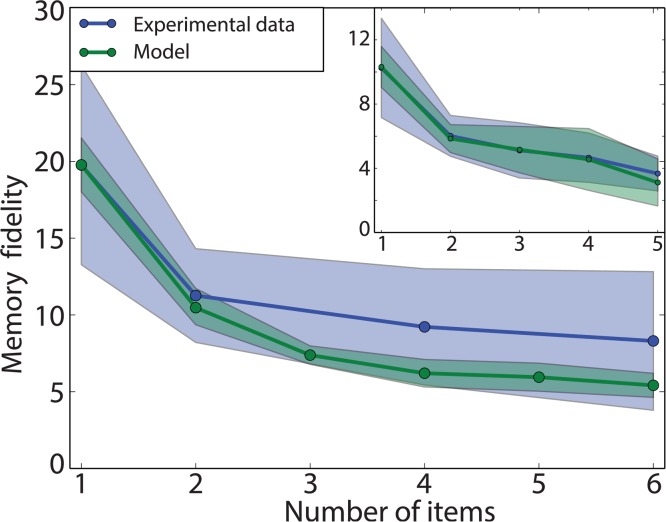
Memory curve fit. Mixed population code. This shows a qualitative fit of the model (green; the shaded area represents one standard deviation) to the human experimental data (blue; data from [7]). *M* = 144, conjunctivity ratio = 0.85, *σ_x_* = 0.1). Inset: similar data fits, for [1] (*M* = 200, ratio = 0.85, *σ_x_* = 0.4). Observe the different decrease in memory fidelity for an increasing number of items.

Fig. 6 shows the fit of our model (in green) to human data (dark blue) from [13], where we report the memory fidelity. The shaded region indicates one standard deviation, computed over multiple reruns of the model (or across different subjects for the human data). The smooth decay in performance as set size increases is appropriately captured by our model. This decay arises in our model from the increase in recall noise as the number of stored items increases, but also from interference between items in the memory. We report in both cases the memory fidelity, the concentration *κ* of the Von Mises component obtained from fitting the mixture model on the responses from human subjects and our model. Here, we used a mixed population code, optimizing the fit to the experimental curve by adjusting the ratio of conjunctive to feature units and the encoding noise *σ*_*x*_, for a population of *M* = 200 units (see Methods for the optimisation procedure). The model does not capture the reduced decay rate for 4 and 6 items to its full extent. However, this is a rather specific characteristic of this dataset. For comparison, the inset in Fig. 6 shows the fit of our model to the data from [1]. In this case, the model captures the memory fidelity dependence more accurately.

### Fisher information analysis

A common theoretical technique used to study the representational capacity of a population code is the Fisher information (FI), which, via the Cramer-Rao lower bound, limits the precision of any estimator based on the output of the code [28–30]. If the posterior distribution in our model can be well approximated as being Gaussian, the FI will accurately characterize memory fidelity, allowing us to examine the effects of different parameters and representations.

In our case, the FI should readily be able to characterise the spread of the errors around the correct target value when a single item is stored (when there is sufficient signal [31, 32]). In this section (and the Supplementary information), we study this case.

When there are multiple items, complexity arises from the fact that errors are distributed around both the target feature value and misbound, non-target, features, with the posterior distribution being multi-modal (and therefore not Gaussian). Nevertheless, as we will see in the next section, the Fisher information, calculated assuming storage of just a single item, can still characterise the memory fidelity around each mode.

Assuming a population code with Gaussian noise and signal-independent noise, the Fisher information is defined as follows:

$${\left[I_{F}\left(\theta \right)\right]}_{ij}={\frac{\partial \mu }{\partial \theta_{i}}}^{T}C^{-1}\frac{\partial \mu }{\partial \theta_{j}}$$ (7)

where ***μ*** is the mean response of the population, and **C** the covariance of the population response. In our case, ***θ*** = [*ϕ* *ψ*]^*T*^, so the Fisher information is a 2-by-2 matrix.

We can easily compute it for the single item case (see Methods), obtaining:

$$\Rightarrow {\left[I_{F}\right]}_{\phi \phi }=\frac{\tau_{1}^{2}}{\sigma^{2}16\pi^{4}I_{0}{\left(\tau_{1}\right)}^{2}I_{0}{\left(\tau_{2}\right)}^{2}}\sum_{i=1}^{M}\sin^{2}\left(\phi -\theta_{i}\right)\exp\left[2\tau_{1}\cos\left(\phi -\theta_{i}\right)\;+2\tau_{2}\cos\left(\psi -\gamma_{i}\right)\right]$$ (8)

In the large population limit in which preferred values have density *ρ*, it is possible to obtain an analytical closed-form solution for this equation which is easier to interpret, (see Section, 1 in S1 Text for the complete derivation)

$$\underset{M\to \infty }{\lim}{\left[I_{F1}\right]}_{\phi \phi }\approx \frac{f\left(\tau_{1},\tau_{2}\right)\rho }{\sigma^{2}}$$ (9)

where *f*(*τ*_1_,*τ*_2_) is an increasing, approximately power-law, function of *τ*_1_ and *τ*_2_ that is given explicitly in the Supplementary information. These values depend on the parameters of the code just as one would expect from classical results for non-circular, uni-dimensional, receptive fields [31, 33]: Increasing the concentration *τ* increases the Fisher information. This is easy to interpret, as narrower receptive fields will be more precise in their encoding of the features. Similarly, increasing the coverage density has the same effect, as more units are available to store information. Finally, the item encoding noise *σ* decreases the Fisher information, as less signal can be extracted from the final memory.

**Figure 7:**
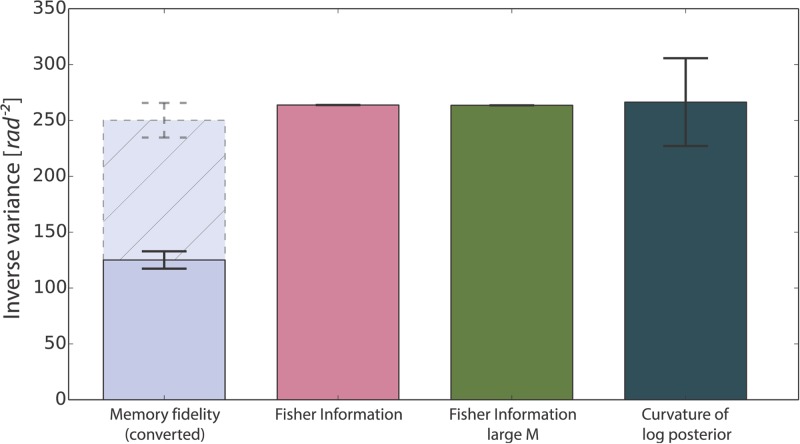
Fisher information fit for one object. Comparison between similar metrics: the memory fidelity (fitted *κ*) of single samples collected for different memory states associated with a single memory state (double the value is shown in dashed blue to take account of the doubly stochastic nature of single sampling); the theoretical Fisher information derived in (8); the large *M* limit for the Fisher information (35); the average inverse variance of samples from the posterior distribution; and the average curvature of the log-posterior at its maximum. This refers to a Conjunctive population code with *M* = 200, *τ* = 4, *σ_x_* = 0.1, *σ_y_* = 10^−5^ and 500 samples.

The Cramer-Rao lower bound transforms the Fisher information into an estimate of performance in the task. Fig. 7 compares the Fisher information for the finite and large population limit with the curvature of the log-posterior at its maximum value (as in the definition of the Fisher information); and to the variance of samples given a memory state. We use again the memory fidelity, by fitting the mixture model onto model samples. We convert this memory fidelity from its units of *κ* into an inverse variance, by converting *κ* to the *σ* ^2^ of the approximated Wrapped Gaussian (see Methods for details). Note that the latter procedure, reporting the variance of samples given a memory state, generates a doubly-stochastic process, hence increasing the variance observed. It can be shown that if the posterior is close to being Gaussian, the variance from those samples will be twice that of the curvature of the log-posterior considered above (see Section, 2 in S1 Text). This is shown by the dashed light blue bar.

We see that they are all similar on average; the most important being the match between samples from our model and the Fisher information analysis.

When more than one item is stored, errors arise from two sources: variance around a mode, and mistakenly reporting the wrong mode (misbinding error). One can adapt the Fisher information analysis to characterize the former, capturing the variability about each mode, conditioned on the fact that the posterior is close to Gaussian. However, it does not capture the component of variance coming from misbinding errors. Further analysis that quantifies both sources of variability will be required to account in a theoretical manner for the full distribution of errors observed in the data.

### Misbinding errors

As noted above, several groups have shown that a significant proportion of the errors made by humans can be explained as arising from “misbinding”, i.e., recalling (at least approximately) the appropriate feature of an inappropriate item, i.e., of a non-target item that also formed part of the array. Such mistakes are shown in Fig. 2B, and could contribute to the appearance of a baseline of errors seen in experiments (Fig. 2A), since these stimuli are drawn randomly from a uniform distribution across all possible angles [7].

The proportion of errors classified as misbinding varies between experiments [1, 7, 13, 34–36]. Although some studies seem to show none at all [37]; in others, they are reported as making up to 30% of all errors when the memory load is high. Misbinding has not been well addressed in the theoretical literature on visual working memory, since current models typically assume distinct subpopulations storing the different items, hence removing any possibility for direct misbinding errors.

Our model uses a single population of units for storage, and so can account for misbinding when the posterior distribution (see Equation 6) becomes multimodal. This usually happens when there is insufficient information in the representation of items to bind the features together (i.e., when the codes are insufficiently conjunctive); the different modes arise from the different items that are stored. The relative heights of the modes of the posterior determine the frequency of misbinding errors. The classical conjunctive population code represents one extreme, offering near perfect binding information, being limited only by the size of each unit’s receptive field. Feature-based population codes, on the other hand, constitute the other extreme: they do not perform binding at all.

**Figure 8:**
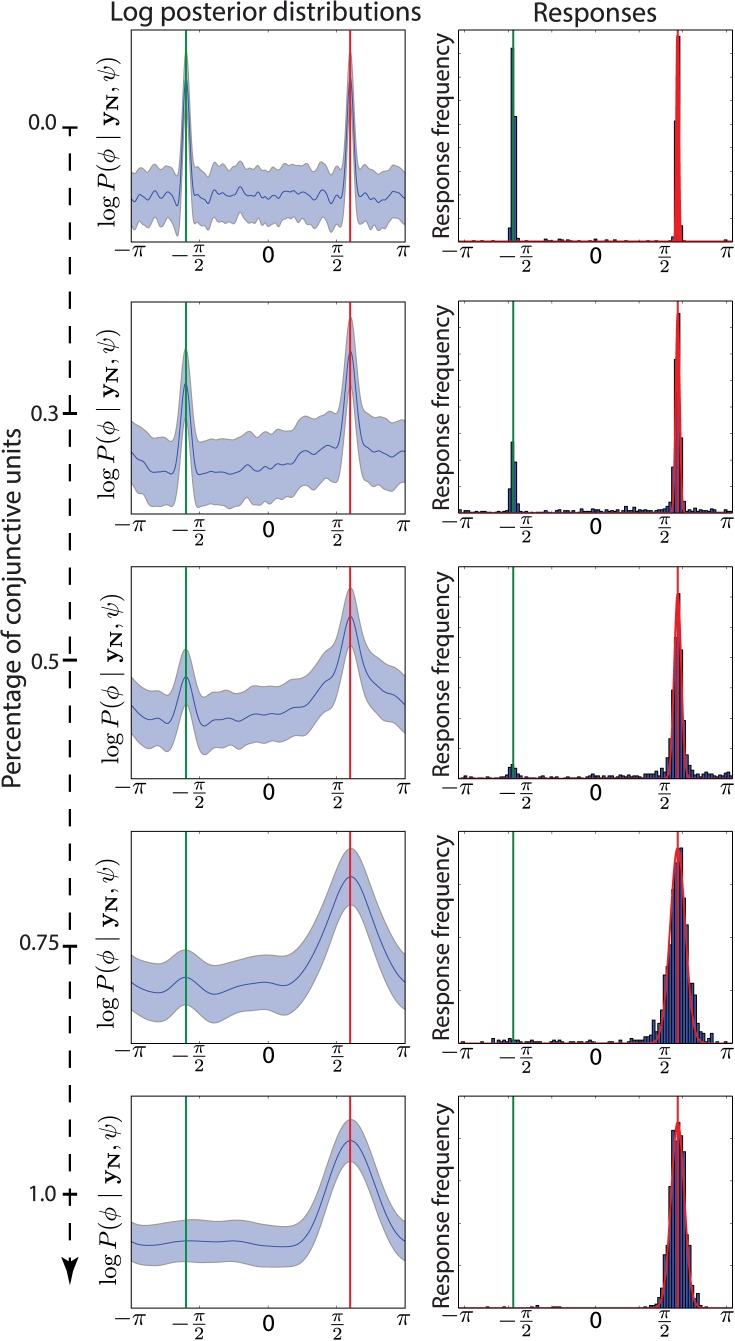
Misbinding errors when varying the proportion of conjunctive units. These plots are based on a mixed population code recalling the orientation of one of two stored items (the correct value is indicated by the red vertical bar). There is a fixed total number of 200 units; the ratio of feature to conjunctive units increases for the graphs going from top to bottom. **Left**: Average (and standard deviation, shown by the penumbra) of the log-posterior distributions over orientation, given the stored memory states averaged over 1000 instantiations of the noise. If the population code only consists of feature units, the posterior comprises two equal modes the incorrect mode disappears as the fraction of conjunctive units increases. However, feature units improve the localization; as their number decreases, the widths of the posterior modes increases. **Right**: Distribution of 1000 sampled responses, showing how misbinding errors tend to disappear when sufficient conjunctive information is available. The red (respectively green) vertical lines indicate the target (respectively non-target) item orientation. The red Gaussian curve shows the probabilit distribution of a Gaussian distribution centred at the correct target value and with a standard deviation derived from the Fisher information of the associated population code.

For a mixed population code, Fig. 8 shows that the proportion of conjunctive units has a strong effect on misbinding errors and posterior multimodality. We construct a situation with two possible angles, $\pm \frac{3\pi }{5}$, where $\frac{3\pi }{5}$ is to be recalled. In this case, using a mixed population code with around 40% of conjunctive units dramatically reduces the number of misbinding errors produced by the model. This proportion will depend on the number of items to be stored, as more items will require more precise binding information.

**Figure 9:**
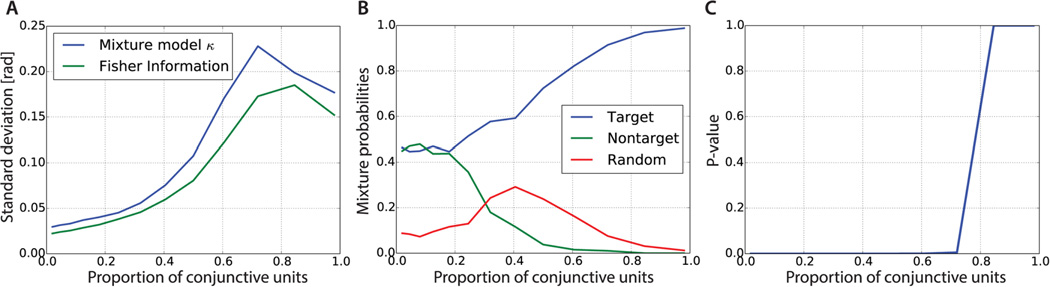
Memory fidelity and mixture proportions as a function of the ratio of conjunctive units. (**A**) Standard deviation of the Von Mises component (in blue) from the mixture model fitted to samples of the model shown in Fig. 8 as a function of the fraction of conjunctive units. The (theoretically-calculated) Fisher information is shown in green for the associated population codes. (**B**) Mixture proportions of the mixture model fitted on the model samples. This metric is less sensitive to random fluctuations of the samples, and shows that if 50% of the units are conjunctive, then 75% of responses will be correctly associated with the appropriate target angle. (**C**) P-value for a resampling-based estimation of the probability of the non-target mixture proportion to be different than zero. We see that the null hypothesis of the non-target mixture proportion being zero can be rejected from 70% of conjunctive units and less.

The widths of the posterior modes depend directly on the amount of information provided by feature and conjunctive units. Feature units are more efficient than conjunctive units at representing single features, and so the cost of reducing misbinding by increasing the proportion of conjunctive units is to increase the width of the posterior over the recalled feature. This can be seen in Fig. 9, where we fitted the mixture model presented in the Modelling visual working memory experiments section to the recall samples, we report the concentration (an inverse width) of the Von Mises component in panel A, and the mixture proportions in panel B.

Fig. 9A confirms the relationships of the width of the posterior mode with the proportion of conjunctive units. The concentration of the Von Mises component (in blue), closely follows the theoretical Fisher information (in green), although overestimating it. The Fisher information provides a good local estimate of the variability around a mode, as can be seen in Fig. 8 on the right, where we overlap in red a Von Mises PDF with a concentration predicted from the Fisher information (with a height set to be aligned with the histogram of the right mode).

The mixture proportions corresponding to the target, non-target and random responses are shown in Fig. 9B as a function of the fraction of conjunctive units. They show that for around 50% or more conjunctive units, more than 75% of responses are on target. The mixture proportion associated with the random component appears to be overestimated, compared visually to the distribution of the samples of Fig. 8. However, the mixture model well characterizes the proportion of misbinding errors.

Finally, as a last check, we verified that the mixture model estimates of non-target proportions were reliable. To do this, we performed a resampling-based analysis of the mixture of non-target responses, by randomizing the assumed locations of non-target angles and re-fitting the mixture model. Using the empirical cumulative distribution over those samples, we could then compute a p-value for the null hypothesis that the mixture probability for non-target would be zero. The results are shown in Fig. 9C, where the p-values as a function of the proportion of conjunctive units in the mixed population code are reported. For proportions of conjunctive units below 70%, the null hypothesis can be rejected significantly (at a 5% level), consistent with the presence of misbinding errors.

We applied this resampling analysis to the human experimental data shown in Fig. 2, as well as to our model’s fit to these data (Fig. 5). The p-values for the data collapsed across subjects are shown above the histograms of biases towards non-target angles; they all are significant. Redoing the analysis per subject indicates that for 2 items, 8 out of 12 subjects show significant misbinding errors; for 4 items, 7 out of 12 are significant; and finally for, 6 items 10 out of 12 subjects show misbinding errors.

### Hierarchical population code

**Figure 10:**
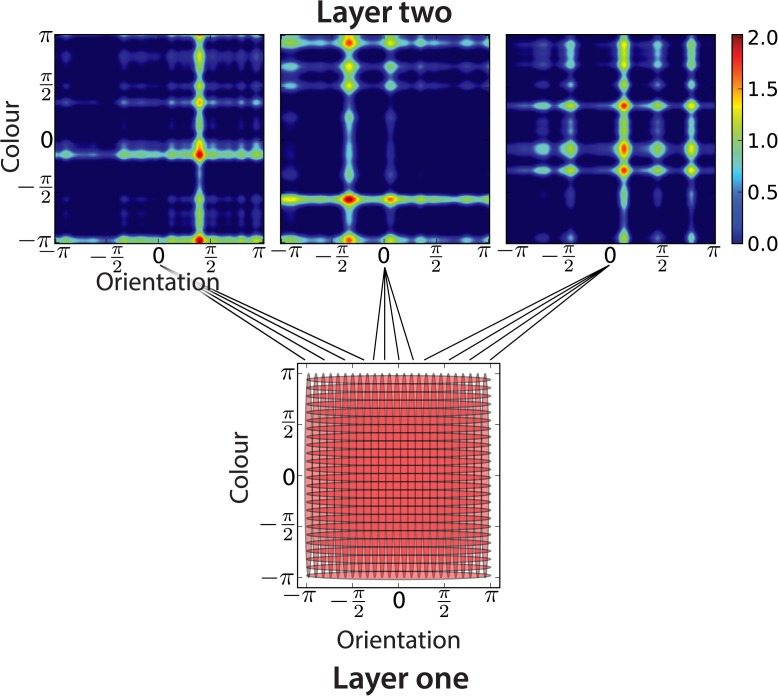
Hierarchical population code. The hierarchical code comprises two layers: the lower layer receives the input, and is randomly connected to the upper one, which provides (possibly additional) binding information. Bottom: layer one consisting of either a feature population code or a conjunctive population code. Receptive fields of units of a feature population code are shown (one standard deviation). Top: effective receptive fields of three layer two units are shown. Layer two units randomly sample a subset of the activity of layer one units, and pass a weighted sum of their inputs through a nonlinearity.

In addition to the “mixed” population code that we have so far described, one might imagine an “hierarchical” population code, shown in Fig. 10. This uses two layers, the lower of which can either be a conjunctive or feature population, parametrised as described above. Units in the higher layer are randomly connected to a subset of the lower layer units, with activities that are a nonlinear (sigmoidal) function of the weighted sum of the sampled units’ activities. More formally, where ***μ*** ^(1)^ is the mean response of the lower layer, *σ*_Θ_ the rectified linear function with threshold Θ:

$$x^{\left(2\right)}\;|\;\phi ,\psi ∼N\left(\sigma_{\Theta }\left(W\cdot \mu^{\left(1\right)}\left(\phi ,\psi \right)\right),\sigma^{2}I\right)$$ (10)

$$\sigma_{\Theta }\left(x\right)=\max\left(0,x-\Theta \right)$$ (11)

$${\tilde{W}}_{jk}∼Bernoulli\left(p\right)\cdot Exp\left(\lambda \right)$$ (12)

$$W_{jk}=\frac{{\tilde{W}}_{jk}}{\sum_{j}{\tilde{W}}_{jk}}$$ (13)

Such an hierarchical code can be considered an abstract representation of a layered neural architecture [38].

The “mixed” and “hierarchical” population codes were specifically introduced to parametrise subtly different forms of binding, controlled by the ratio of binding to non-binding units. In the “mixed” population code, conjunctive units introduce binding information independently from the rest of the feature units. In the “hierarchical” population code, the random layer two units bind together the activity of layer one units, generating seemingly arbitrary combinations of feature values, yet providing sufficient conjunctive information. It allows us to check how structured the binding information should be for the results to hold.

**Figure 11:**
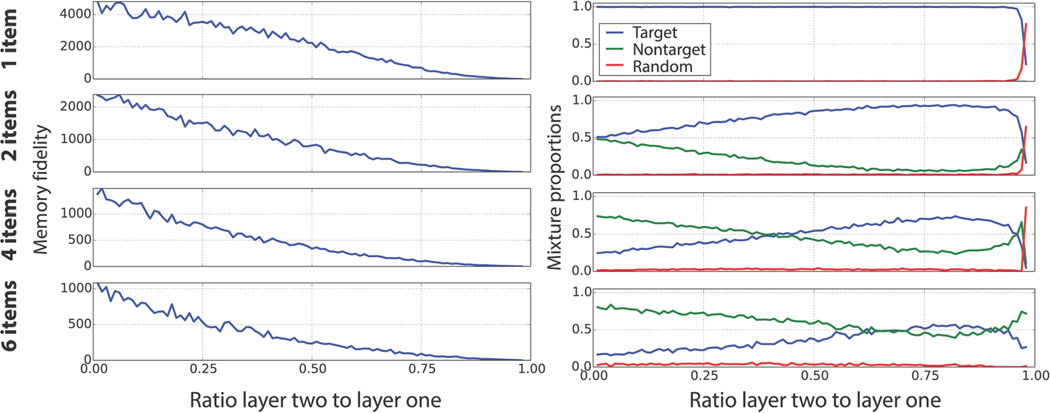
Memory fidelity and misbinding errors as function of conjunctivity in hierarchical population code. **Left**: Memory fidelity based on model samples, while varying the ratio of lower to upper layer units in a hierarchical population code with a constant number of 200 units. The number of (randomly placed) items increases from top to bottom. The memory fidelity decays with increasing item number and conjunctivity. **Right**: Mixture proportions based on model samples. For a single item, the correct target angle is always retrieved (blue curve). The drop for high ratio of upper to lower layer is expected, as few units are left in the lower layer to represent the item appropriately. For increasing numbers of items, nontarget responses are prevalent (green curve), but including a suitable proportion of upper layer units does allow the appropriate angle to be retrieved. Random responses are marginal with the parameters used here. *M* = 200, *σ_x_* = 0.2.

Fig. 11 shows the behaviour of recall for a hierarchical population code based on a feature population code at the lower layer. The total number of units was fixed (at *M* = 200); the ratio of upper to lower units was varied. The optimal arrangement changes markedly when multiple items must be stored. Having few random binding units is very efficient in the single item case, but this breaks down completely when multiple items are stored and interfere with each other. The dependence of the memory fidelity on the ratio of upper to lower units is similar for increasing number of items, with the exception of the overall scale. Unsurprisingly, memory fidelity is lower when increasing the number of items and conjunctivity, see Fig. 11A. As shown in Fig. 11B, the probability of the response being related to the correct target changes completely going from one to many items, with non-target responses becoming prevalent for small ratios of upper to lower units. Moreover, there is an optimal ratio of upper to lower units when storing multiple items, if one tries to optimise the proportion of correct target angle recall.

**Figure 12:**
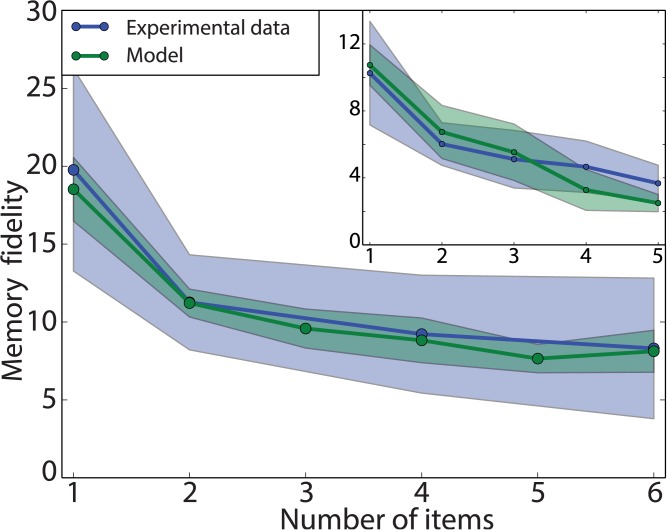
Memory curve fit for hierarchical population code. Model fit (green; the penumbra represents one standard deviation) to the human experimental data (blue; data from [7]). These qualitative fits are similar to those obtained for a mixed population code (see Fig. 6), despite the significantly different implementation. (*M* = 200, ratio = 0.9, *σ_x_* = 0.3). Inset: fit for [1]. Notice the difference in performance for large number of items. (*M* = 200, ratio = 0.9, *σ_x_* = 0.55)

Fig. 12 shows the fit of the memory fidelity to the experiments in [1, 7], as was done in Fig. 6 for the mixed population code. Despite being drastically different in its implementation of conjunctivity, it provides a good fit to the experimental data. The hierarchical code is able to capture the trend of decay in both experiments to a greater extend than the mixed population code (main plot shows a fit to [7], inset shows a fit to [1]). However, the fit for 4 and 5 items for [1] does show discrepancies with the experimental data. The optimal parameters obtained for those fits resemble those for the mixed population code, namely a high ratio of higher-level binding units and large input noise. These render promising this class of hierarchical codes.

### Comparisons of population codes

#### Effects on experimental data fits

The patterns of errors arising from specific choices of population codes can be used to help discriminate between different representations. Misbinding, which we quantify via the mixture model approach of [7], is of particular value, since, as observed, it is rare for conjunctive codes; but ubiquitous for feature codes. We therefore compare the misbinding exhibited by human subjects with the output of our model based on different population codes (see Methods for details about the optimisation).

**Figure 13:**
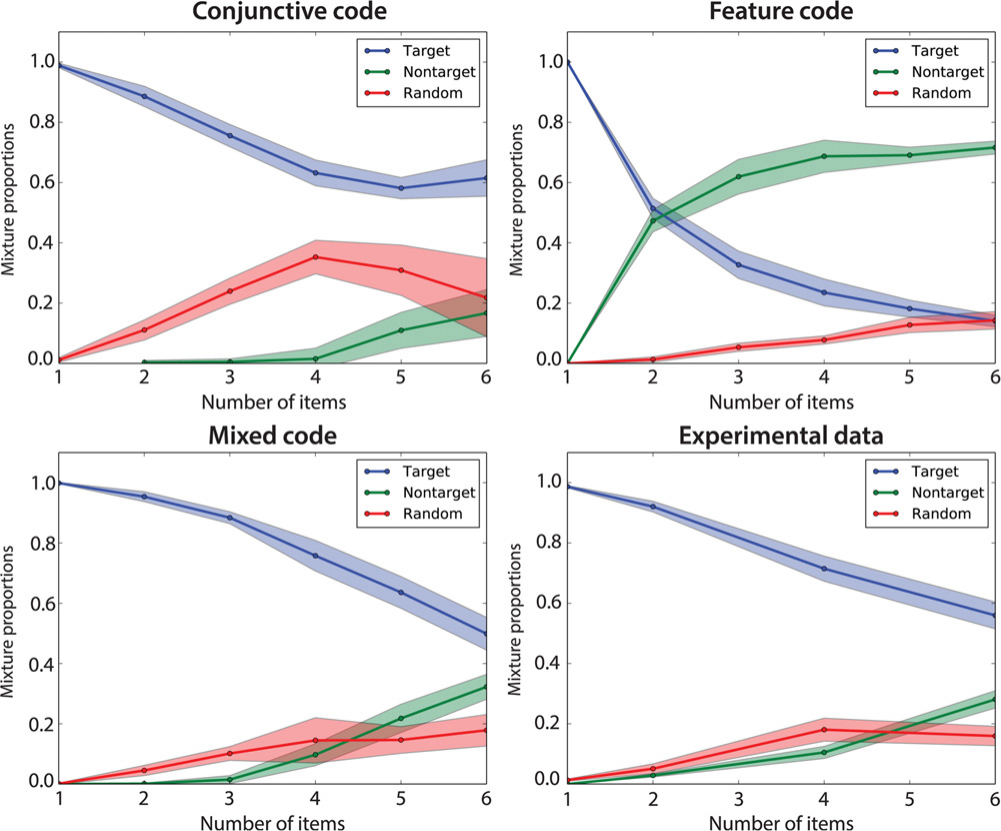
Error types for different population codes. The graphs quantify different sorts of error in terms of the weights in a mixture model capturing local variability around an item, misbinding errors and random choices [7]. Human experimental curves are shown on the bottom right. This shows how misbinding errors are crucial components to fit human performance. Conjunctive population code: *M* = 225 units, *σ_x_* = 0.3, Feature population code: *M* = 100 units, *σ_x_* = 0.08, Mixed population code: *M* = 144 units, conjunctivity ratio = 0.85, *σ_x_* = 0.1

As can be seen in Fig. 13, there are clear differences in the mixture weights associated with misbinding errors, errors arising from local deviations from the correct feature to be recalled, and the uniform component.

As expected, the feature code makes a large number of misbinding errors when more than one item is stored. On the other hand, the conjunctive code makes only a few errors that appear to arise from random guesses. Misbinding errors are highly unlikely in this configuration. In total, a mixed code provides a better fit to the human data, matching the increase in non-target responses as well as a baseline random response rate.

#### The canary

**Figure 14:**
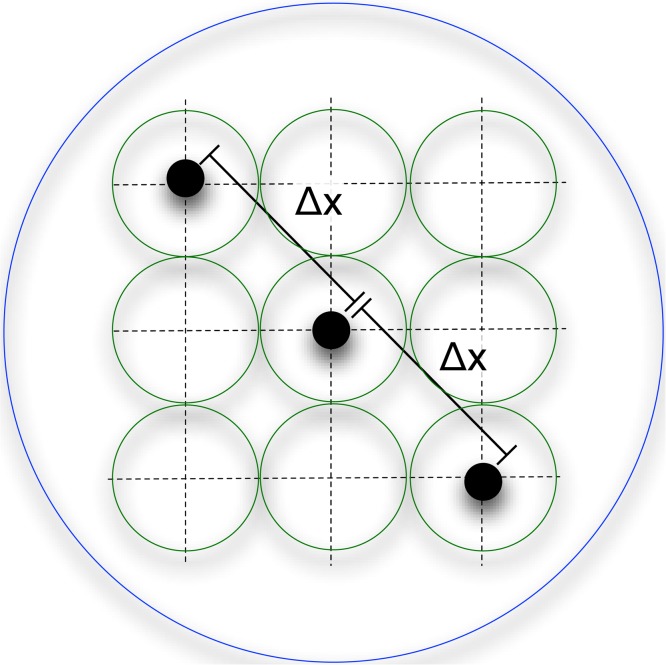
Stimulus pattern to induce misbinding. Feature-space representation of three stimuli used to study misbinding errors and characteristics of the population codes. Three items are separated by a distance Δ*x*. This set of items will generate interference patterns as shown by the dotted lines. The circles represent one standard deviation of the receptive field response levels. The green circles represent a population code in which the three stimuli are well separated. The blue circle represents a code for which all the stimuli lie inside a single receptive field and would generate misbinding errors. The target is randomly chosen on each trial as one of the three items.

This analysis suggests that stimuli specifically designed to induce patterns of misbinding could be useful for understanding representations in population codes. Consider three stimuli, arranged on a diagonal, separated by a variable distance in feature-space (illustrated in Fig. 14). These create clear interference patterns for feature codes, with multi-modal posteriors and misbinding errors. These errors will be expected to change as a function of the characteristics of the population code. We therefore call this stimuli pattern the “canary” in honour of its capacity to reveal such characteristics.

In particular, by making the stimuli close to each other in feature space, this pattern allows intra-receptive field misbinding to be examined. This happens when the pattern lies entirely in a single receptive field of a conjunctive unit, and can thus provide a somewhat crude and indirect measure of the receptive field size of a mixed conjunctive code. Note, though, that hierarchical conjunctive codes cannot be expected to have such a simple signature; and indeed even mixed codes are ultimately likely to be multi-scale in character.

**Figure 15:**
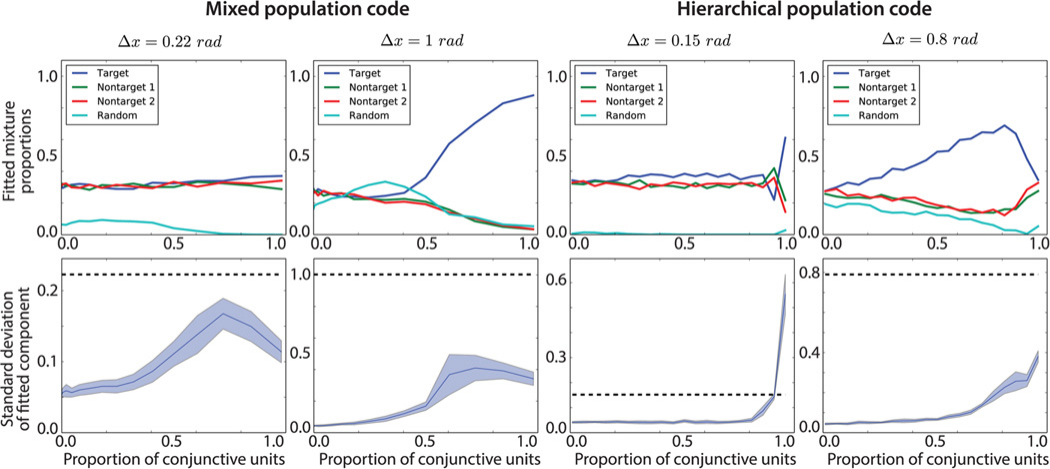
Recall of stimuli shown in Fig. 14. 100 individual samples from the model are generated for specific parameters (*M* = 200, *σ_x_* = 0.25), mixed (left) or hierarchical (right) population codes and inter-stimulus distances Δ*x* = {0.22, 1} rad. Shaded regions correspond to one standard deviation around the mean over 10 repetitions. **Top row**: Fitted mixture proportions from a mixture model (with one Von Mises component per target/non-target and a random uniform component, similar to [7]). For small Δ*x*, no amount of conjunctivity can improve the results, indicating intra-receptive field misbinding. For large Δ*x*, there is a change from non-target to target responses as the proportion of conjunctive units increases. The target is randomly chosen for each trial.. **Second row**: Width of the Von Mises component of the mixture model (represented as the standard deviation corresponding to the fitted concentration *κ*). The dotted black line corresponds to the distance Δ*x* between items in the stimuli pattern.

In Fig. 15 (left panels), we show what happens for a mixed population codes. We report how the parameters of the mixture model we considered before vary with conjunctivity in several conditions, using a population code of 200 units, and allowing the ratio of conjunctive to feature units to vary from 0 to 1 (corresponding to full-feature and full-conjunctive, respectively). We set the item noise *σ*_*x*_ = 0.25, a level compatible with experimental data fits, and show two characteristic distances between stimuli, Δ*x* = {0.22,1} rad. The goal is to recall one of the three items, randomly chosen on different trials. We characterize the errors using the mixture model presented before and report the mixture proportions and the fitted *κ* from the Von Mises component.

For the large separation, Δ*x* = 1.0, the mixed population code behaves in a regular manner as the degree of conjunctivity increases. For a feature-based population, recall suffers from much misbinding; it is only when more than 50% of the units are conjunctive that correct binding typically occurs. The mixed population code increases rapidly at around 70% of conjunctive units and saturates.

The outcome is quite different for the small separation Δ*x* = 0.2. In this case, no amount of conjunctivity can help the discrimination between the three stimuli. This corresponds to a situation in which intra-receptive field misbinding occurs. Even for a fully conjunctive population code, the size of the receptive field is larger than the distance between two items (*M* = 200, *τ* = 5.5 ⇒ width of 0.44 *rad* for one standard deviation of a receptive field).

**Figure 16:**
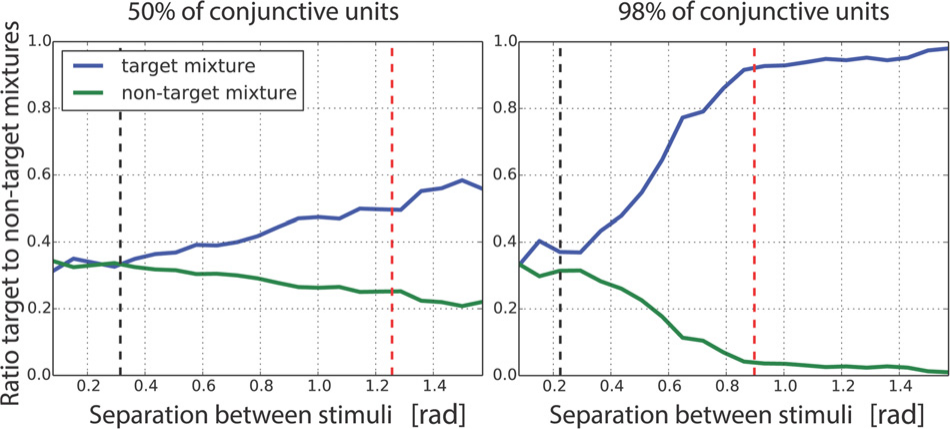
Patterns of errors as a function of stimulus separation for different proportions of conjunctive units. This shows data as in Fig. 15, but as a function of the varying distances in radians between stimuli in the diagonal pattern, for two mixed populations with 50% and 98% conjunctivity. We compute the ratio between the target mixture proportion and the sum of the target and non-target mixture proportions (in blue). We do the same for a non-target mixture proportion (in green). The black vertical bars show half the size of a conjunctive receptive for each population. We see that for separations smaller than the size of a receptive field, misbinding errors are prevalent. This changes as soon as the pattern of stimuli covers more than one receptive field. The vertical red dashed bar shows twice the size of a receptive field. In this situation, each stimulus occupies one receptive field, and misbinding should rarely occur.

For the single-scale receptive fields that we employed to create the mixed population code, it is possible to recover the scale from the error patterns as a function of the separation between the stimuli. This is shown in Fig. 16 for two mixed population, with 50% and 98% of conjunctive units. This plots the target (blue) and non-target (green) mixture probabilities (normalized by their joint sum). These start at the same value, but diverge after the point when conjunctive information becomes available and hence when intra-receptive field misbinding become less prevalent. The black vertical dotted line indicates half the size of the receptive field for the conjunctive subpopulation—misbindings stop being prevalent once the stimuli covers multiple receptive fields. The red line for the case of 98% conjunctive units corresponds to two times the size of the receptive field for the conjunctive subpopulation. Once this point is reached, each stimulus lies in its own receptive field, so misbinding should not happen. This is again in agreement with the results, with very few non-target responses in this regime.

We originally expected a hierarchical population code to perform differently, since it encodes binding information in a quite different manner. However, surprisingly, we find consistent results, as can be seen in Fig. 15 (right panels). Again, we show two characteristic distances between stimuli in the canary pattern, Δ*x* = {0.15,0.8} rad (we chose different values than in the mixed code situation, as the population codes behave slightly differently).

When the separation is large, the hierarchical code also behaves in a regular fashion similar to that of the mixed code as the degree of conjunctivity increases. When conjunctivity is low, the memory performs poorly, as no binding information is present. However, as conjunctivity increases, performance does as well. Interestingly, performance with a hierarchical code increases monotonically with conjunctivity (before dropping sharply when the input lower layer population decreases past the required precision needed to discriminate the stimuli). This architecture uses conjunctive information quite effectively, but does not attain the same maximum performance.

The situation is less clear for a small distance between stimuli. Having a large proportion of conjunctive units is actually detrimental in this case, as the input lower layer decreases in size, and thus the encoding precision decreases with it. Hence there is an optimal proportion of conjunctive units for a given required minimum discrimination. The smallest distance for which the target and non-target responses can be discriminated when analysing the results is Δ*x* ≈ 0.30, using a hierarchical code with a conjunctivity of 80% (see Section 3 in S1 Text). Hence the hierarchical code seems to discriminate smaller patterns for a given population size, which is surprising for such a crude representation of a hierarchical representation.

Thus we find that even this simple stimulus pattern can provide something of a formal window into misbinding and the structure of receptive fields.

## Discussion

We built a model of short-term visual working memory, assuming a single population of units, an additive, palimpsest, storage scheme and sample-based probabilistic recall. We showed how this model could qualitatively reproduce key aspects of human experimental data, including the decrease in performance with memory load, and also error distributions, including misbinding errors, which have not previously been the focus of theoretical study. It is the next phase of this work to fit human data quantitatively, looking in detail at individual differences in performance and patterns of errors.

We studied several different sorts of population code. The most critical question concerns binding, which in our case is performed by conjunctive units that are sensitive to specific combinations of two or more features. Non-conjunctive, feature-based codes, can be more efficient at storing single items, but fail catastrophically whenever multiple items are stored simultaneously. We considered including both single-feature and conjunctive units, and showed that a combination is likely to offer a better characterization of experimental data than either alone. Finally, we considered experiments that would offer useful guidance to discriminating theories.

The original such model of this class of experiments was formalised by Wilken & Ma [39], based on experiments and arguments from Pashler and Luck & Vogel [4, 40]. This includes a finite set of “slots”; items that are not allocated a slot are therefore not remembered at all (requiring pure guessing for recall). The assumed error distribution was thus a mixture model with two components: a Von Mises centred around the target item, and a random uniform component. The alternative models are based on the notion of a finite resource [1, 7–11], arguing against a fixed number of slots, but rather that there is a constraint on the whole collection, such that storage of multiple items leads to interference. More recently, intermediate accounts have been suggested, such as a “slots and averaging” model [5], letting individual items be stored in more than one slot, with the outputs of all the slots concerned being averaged.

By comparison, our model, as a palimpsest, can best be seen as abandoning the notion of slots altogether—be they finite or infinite—and so does not need a mechanism for allocating the slots. There is a finite resource—the population of units that can be active—but this leads to two resource-like limitations on storage, rather than one. The first limitation is noise—this acts just like some of the resource limits in previous models. The second limitation is representational—the fact that the items overlap in the palimpsest in a way that depends on how they are encoded in the population implies a form of interference and interaction that leads to misbinding. This explicit element has been missing in previous treatments. Along with the variability in the process of sampling, it is key to the model’s account of the pattern of errors of human subjects, with heavier tails than a Gaussian/Von Mises distribution. Other factors have also been implicated in this pattern, such as different memory encoding precision on different trials [10, 41], or the limited width of neuronal tuning functions [15]. It would be straightforward to extend our scheme to allow for partial information about which item will have to be recalled.

We have shown how our model can encode information about each feature separately, with the binding information being provided by another subpopulation. A model along related lines was recently proposed by Swan and Wyble [42]. In this, an associative network, which they call the “binding pool”, provides binding information. However, one could think of other ways to encode and store this binding information, for example by using object-files. If one were to limit how many object-files could be used at a given time, and if object-files made errors in binding the features together, this would provide an hybrid slot-based treatment of the problem.

Another related model has been suggested in the context of dynamic field theory [43, 44]. These authors consider a population of rate-based units with temporal dynamics governed by first order differential equations. Given specific layers and connectivity patterns, they simulate the evolution of bumps of activity through time, which can be used to store information for later recall. In their model, feature binding is completely linked to space in that each feature is stored in different feature-space population bound only to location. A separate working memory population stores the locations of all items seen. Recall relies on using location to couple and constrain the possible features to their original values. This idea resembles “feature integration theory”, proposed by [45] as a model for attention.

That the dynamical (e.g., drifting) behaviour of the bumps is the critical focus of the model sits a little uneasily with the observation that performance in visual short-term memory experiments does not drop significantly when recall is delayed [1, 46]. Further, location cannot be the only variable determining binding given experiments in which items are presented at the same location but at different times. Our model is agnostic about the source of binding in its input, lending itself to the study of different representations. Nevertheless, it would be interesting to model richer aspects of the temporal evolution of the memory state.

Here, we assumed that only two features were stored per item, namely colour and angle. However, we report in Section, 5 in S1 Text the effect of using more than two features. One feature that is particularly important is spatial location. In the actual experiments in [7], space (which, for simplicity and consistency with [1], we treated as another angular variable) was used as the cueing feature, with colour being recalled. It is possible, given the importance of space for object recognition, that spatial tuning has quite different characteristics from that of other cues. Hints of this are apparent in the properties of early visual neurons. This could make it a stronger cue for recall and recognition, something that it would be interesting to examine systematically through experiment and the model.

With more features, we could address directly one of the key findings that led support to the slot models, namely the observation of an object benefit in recalling features. That is, despite the sometime fragility of episodic memory [47], which this functionally resembles, remembering a fixed number of features is easier when those features are parts of fewer conjunctive items. The magnitude of that effect has been the subject of intense debate, but there is broad agreement about a significant object benefit [48–52]. In our model, such effects arise through two mechanisms: first, having fewer items will add less encoding noise to the final memory state, which will directly reduce the overall noise level in recall. Second, the conjunctive units also directly contribute to the storage precision for bound items. Our model would thus also show an object benefit without additional machinery.

Our model treats storage as a bottom-up, feedforward process. However certain top-down effects are known, such as directed forgetting [53, 54]. Such an effect could be accommodated in the model by considering a multiple step process in which following regular storage, recall would be executed based on the cue for the to-be-forgotten item, with the representation of whatever is retrieved being subtracted from the previous memory state. As this would still be a noisy process, the resulting precision for the other items would be less than if the forgotten item had never been stored at all, albeit still greater than if its main influence over the memory state remained.

We made a number of simplifying assumptions, notably to do with the noise model and the sampling process. For the former, we only considered additive isotropic Gaussian noise corrupting the encoding. This could be readily extended to more complex noise models, for example to a more neurally plausible Poisson noise model. The key difference from using Poisson noise would be its signal-dependence—storing larger numbers of items would lead to greater activities and thus a higher variance. Signal-dependent Gaussian noise is a related modelling choice [30, 31, 55]. Amongst other differences, this would reintroduce the second term in the equation for the Fisher information (Equation 30). This term can be large compared to the first [55] and it adds extra inferential complexity [56], hence fully accounting for it can be complicated.

We considered a process of recall that involves the full posterior distribution over the responses. Determining how the brain would use and represent distributional information has been an active recent research topic. One set of ideas considers what amounts to a deterministic treatment (albeit corrupted by noise) [57–62]. However, there is a growing body of research showing how the brain might instead use samples [63–66], and we adopted this approach. Inference might involve combining together larger numbers of samples, and thus reporting some (noisy) function of the posterior other than just the samples. However, such operations are currently underdetermined by the experimental data, as they would interact with other sources of noise. Sampling from the posterior instead of simply reporting the maximum a-posteriori mode value has the additional benefits of capturing variability around the mode itself, which varies depending on the representation used. Nevertheless, it is important to stress that this sampling scheme is not the main bottleneck in our model. Rather, it is the representation that constrains the nature and magnitude of the errors in recall. The sampling scheme simply provides a mechanism for reporting on the ultimate posterior distribution. A more limited report, such as the MAP value, would likely lack the appropriate characteristics by reflecting too little of this distribution.

One of the major tools that we used to analyse the population codes was the Fisher information (and the associated Cramer-Rao lower bound). However, this is only useful if the posterior distribution is close to being Gaussian, and, in particular, unimodal. This will almost always be true for a single item; and often be true when there are multiple items and a conjunctive population code that solves the binding problem. However, as we saw, feature codes lead to multimodality, rendering a direct application of the Cramer-Rao lower bound useless. What is still possible is to use the Fisher information as an indication for the variability around one of the mode. We have shown how it still produces a good approximation to the width of a mode, even in the presence of misbinding errors.

We characterized misbinding errors through a mixture model and a resampling-based estimator. It is also possible to assess the multimodality of the posterior itself directly, for example by fitting a parametric mixture model on the posterior. This analysis leads to similar results. But it would then be possible to analyse this multi-modality analytically, and perhaps obtain a closed form expression for the proportion of misbinding errors expected from a given posterior.

We considered a case of recalling only a single item given a memory. It would be possible to treat recall differently, with a mixture model, estimating the features associated with all items, and thereby answering the memory query directly. Total recall could be performed using a fixed finite mixture model, e.g. a Gaussian Mixture model, but lends itself well to a nonparametric extension, characterizing the whole collection of elements in an array. Approaches of this sort have been pursued by various recent authors [67–71]. For instance [71] considered both the encoding and recall to be implemented with a Dirichlet process mixture model. They show how this provides a natural account of ensemble statistics effects that can be seen in some experiments, such as regression to the mean of the presented samples. By contrast, our approach is closer to the experimental paradigm, as there is no evidence that subjects recall all features of all items when asked to recall an unique item. Regression to the mean still arises, but from local interactions between items in the representation. Indeed, even for a conjunctive code, when items are close-by the recalled angle will be biased towards the mean of all items, as bumps of activity merge together. There is substantial precedence for the approximation of focusing on a single item, ignoring some or all of the statistical structure associated with other actual or potential items [72–75].

Our results depend crucially on the nature of the underlying population code. As a proof of principle, we tested two schemes—one mixing feature-based and conjunctive codes; the other building a hierarchy on top of feature codes. However, many more sophisticated representations would also be possible—studies of population coding suggest that using multiple scales is particularly beneficial [76, 77], and it would be interesting to test these.

For our single-scale case, we suggested a particular pattern of three stimuli that we expect to be of particular value in discriminating between different population coding schemes. The pattern was designed to promote misbinding in a way that would also be revealing about the size of the receptive fields. We also expect there to be a strong effect of distance in stimulus space on misbinding probability, if a mixed-like representation is used. On the other hand, by the very nature of our hierarchical population code, it is harder to make specific predictions about the dependence of proximity and other features on misbinding probability. If subjects were too proficient at recall from this pattern, as might be the case for just three items [1], it would be straightforward to complicate the scheme to include a larger number of items.

An interesting extension to this analysis would be to introduce an asymmetry in the pattern of stimuli, in order to displace the mean of the stimuli from the centre stimulus. This would in turn introduce asymmetric biases and deviations for the different items depending on the sources of the errors. Indeed, as briefly mentioned above, it has been shown that the mean statistics of the stimuli have an effect in determining responses characteristics. Such an asymmetric pattern would indicate if the variability is biased towards the mean of the stimuli or to close-by items only.

Although our proposal has primarily been grounded on the psychophysical literature, the use of population representations, and the abandonment of anatomical “slots”, makes it appealing to consider the neural basis of the memory. There is substantial work on population-based working memory with a foundation in persistent activity [78], and even in the gating of storage necessary to make such memories work efficiently [79, 80]. It would be interesting to study the extra constraints that come from a more realistic neural implementation.

In conclusion, we proposed a model which accounts for errors in working memory by considering explicitly the link between storage and representation. We showed it can successfully account for key aspects of the psychophysical data on visual short term memory, and allows for a better understanding of the relationship between being precise in the representation of single features and the representation of binding information across all the features of a single pattern to be able to handle cued recall. Based on observations on the form of the errors arising when recalling information from a palimpsest memory, we proposed a specific stimulus template that would produce different error patterns depending on characteristics of the underlying representation, and so we suggest as an attractive target for psychophysical investigation.

## Methods

Here, we provide a complete description of the processes of storage and recall, repeating material from the main text as appropriate for convenience.

### Representation

We assume continuous firing-rate style units. They have Bivariate Von Mises tuning curves, corrupted by isotropic additive Gaussian noise:

$$\mu_{m}\left(\phi ,\psi \right)=\frac{1}{4\pi^{2}I_{0}\left(\tau_{1,m}\right)I_{0}\left(\tau_{2,m}\right)}\exp\left(\tau_{1,m}\cos\left(\phi -\theta_{m}\right)+\tau_{2,m}\cos\left(\psi -\gamma_{m}\right)\right),$$ (14)

*ϕ* and *ψ* are respectively the orientation and colour of the item to be represented. *θ*_*m*_ and *γ*_*m*_ identify the preferred angle and colour of unit *i*. *τ*_1,*m*_ and *τ*_2,*m*_ control the size of the receptive field, as well as the sensitivity of each unit to the different features.

Let the population firing rate state be **x** = [*x*_1_,…,*x*_*M*_]^*T*^, *x*_*m*_. The firing rate of unit *m* is:

$$x\;|\;\phi ,\psi \;∼\;N(\mu \left(\phi ,\psi \right),\;\sigma_{x}^{2}I)$$ (15)

Differences in the choices of *τ*_1,*m*_ and *τ*_2,*m*_ across the population will generate different types of representation.

The hierarchical population code is defined as follows, with ***μ*** ^(1)^ being the mean response of the lower layer.

$$x^{\left(2\right)}\;|\;\phi ,\psi ∼N\left(\sigma_{\Theta }\left(W\cdot \mu^{\left(1\right)}\left(\phi ,\psi \right)\right),\sigma^{2}I\right)$$ (16)

$$\sigma_{\Theta }\left(x\right)=\max\left(0,x-\Theta \right)$$ (17)

$${\tilde{W}}_{jk}∼Bernoulli\left(p\right)\cdot Exp\left(\lambda \right)$$ (18)

$$W_{jk}=\frac{{\tilde{W}}_{jk}}{\sum_{j}{\tilde{W}}_{jk}}$$ (19)

The receptive field sizes were set automatically to achieve maximum coverage given a population of *M* units. Given a fixed number of units with preferred stimuli arranged uniformly over the feature space, the receptive field sizes were modified such that one standard deviation of the receptive field would cover the space uniformly without redundancy.

In the case of a conjunctive code, we have:

$$\tau =g_{\sigma \to \tau }\left(\frac{2\pi }{\sqrt{M}}\right)$$

where *g*_σ → τ_ converts the standard deviation of a Wrapped Gaussian into the τ of a Von Mises. No closed-form solution of *g*_σ → τ_ exists; it can be computed numerically by finding the argmin_τ_ ${\left(\exp(-\frac{\sigma^{2}}{2})-\frac{I_{1}(\tau )}{I_{0}(\tau )}\right)}^{2}$.

For a feature code, we set:

$$\tau_{1}=g_{\sigma \to \tau }\left(\frac{2\pi }{M/2}\right)$$ (20)

$$\tau_{2}=g_{\sigma \to \tau }\left(2\pi \right)$$ (21)

Where *τ*_1_ and *τ*_2_ correspond to the two receptive field sizes of one subpopulations (here assumed to be sensitive along the *τ*_1_ direction).

### Storage and recall process

The storage process for *N* items is probabilistic and follows the following model:

$$x_{i}\;|\;\phi_{i},\psi_{i}\;∼\;N\left(\mu \left(\phi_{i},\psi_{i}\right),\sigma_{x}^{2}I\right)$$ (22)

$$\;y_{N}\;|\;x_{1},\;...\;,x_{N}\;∼\;N\left(\overset{N}{\underset{i=1}{\sum }}\beta_{i}x_{i},\;\sigma_{y}^{2}I\right)$$ (23)

**x_i_** is the representation of item *i* by the population code. *ϕ*_*i*_ and *ψ*_*i*_ represent the feature values of item *i*. Multiple items are summed to produce the final memory state **y_N_**, which is, in turn, corrupted by additional, independent, Gaussian, noise. *β*_*i*_ models different strengths of storage in the memory (to accommodate tasks involving explicit attentional instructions).

Recall is based on the simplifying assumption that a single item is modelled, while others are collapsed into a single source of noise. **m_N-1_** is the contribution of the noise process to the mean of the final memory state and Σ_**N**_ is the contribution of the noise to the full memory covariance. *r* is the index of the item to be recalled, which we integrate over as it is unknown during recall. The posterior over the feature *ϕ* to be recalled is defined as follows:

$$y_{N}\;|\;\phi ,\psi ,r\;∼\;N\left(m_{N-1}\;+\;\beta_{r}\mu \left(\phi ,\psi \right)\;,\;\Sigma_{N}\right)$$ (24)

$$\phi \;|\;y_{N},\psi \;∼\;\int^{\text{​}}dr\;p\left(r\right)p\left(\phi \right)\;p(y_{N}\;|\;\phi ,\psi ,r)$$ (25)

We use uniform prior distributions over *r* and *ϕ* (circularly uniform for *ϕ*).

The collapsed noise mean **m_N-1_** and covariance Σ_**N**_ can be estimated from random samples of the storage process. **m_N-1_** is the mean memory built from *N*−1, marginalising over feature values:

$$m_{N-1}=E\left[y_{N-1}\right]$$ (26)

$$y_{N-1}\;∼\;\underset{\phi_{1},\psi_{1}...\phi_{N-1},\psi_{N-1}}{\int^{\text{​}}...\int^{\text{​}}}P(y_{N-1}|\phi_{1},\psi_{1},\cdots \phi_{N-1},\psi_{N-1})d\phi_{1}d\psi_{1}\cdots d\phi_{N-1}\psi_{N-1}$$ (27)

Similarly, Σ_**N**_ is the covariance of *N* items, marginalising over feature values. We obtain estimates by sampling 5000 memory items from the storage process before estimating those two empirical estimates.

We use a slice sampling scheme to obtain samples of *ϕ* given a memory state. In addition to the classical slice sampling algorithm, we introduce Metropolis-Hastings jumps, which can randomly set the sampler in another part of the state space. This allows to jump between modes in a multi-modal posterior setting. The jump probability is set to 10% and a jump is accepted depending on a Metropolis-Hastings acceptance ratio. We discard the first 500 samples as burn-in steps for the slice sampler. We perform step-out and shrinkage to determine the slice width (initially set to $w=\frac{\pi }{40}$) [26]. We constrain the sampler to the [−*π*,*π*] interval. This allows us to sample appropriately from the full posterior.

### Mixture model fitting

We use the mixture model of [7], allowing for a mixture of target, non-target and random responses. We fit the following mixture component, using the expectation-maximization algorithm:

$$P\left(\theta \right)\;=\;p_{t}VM\left(\theta ;\mu_{t},\kappa \right)+\overset{N-1}{\underset{k}{\sum }}p_{nt}VM\left(\theta ;\mu_{k},\kappa \right)+p_{r}\frac{1}{2\pi }$$ (28)

$$p_{t}+p_{r}+p_{nt}=1$$ (29)

where *p*_*t*_ is the mixture proportion associated with the target, *p*_*r*_ the random mixture proportion and *p*_*nt*_ the non-target mixture proportion. *μ*_*t*_ and *μ*_*k*_ are the true locations of the target and non-targets. All Von Mises share the same *κ*; this is because the concentrations (though not the mixing proportions) of the posterior modes around each target are determined by the Cramer-Rao lower bound associated with the local Fisher information, which are all identical. The values of *p*_*t*_,*p*_*r*_,*p*_*nt*_ and *κ* are fit during the EM procedure; the *μ*’s are assumed to be known.

To check for the significance of non-zero mixture proportion *p*_*nt*_, associated with non-target responses, we perform a resampling analysis. Given a set of responses, targets and non-target angles, we randomly resample the non-target angles and refit the mixture model. We perform this procedure *K* times and obtain *K* samples of *p*_*nt*_ (*K* = 1000). We then construct the empirical cumulative distribution function Φ(*p_nt_*) for *p_nt_* given those samples. Finally, we compare the mixture proportion $p_{nt}^{*}$ obtained given the original non-target angles, and reject the null hypothesis “*p*_*nt*_ = 0” when $p=1-\Phi (p_{nt}^{*})<0.01$.

### Fisher information derivation

The Fisher information for a population code with Gaussian noise is:

$${\left[I_{F}\left(\theta \right)\right]}_{ij}={\frac{\partial f}{\partial \theta_{i}}}^{T}C^{-1}\left(\theta \right)\frac{\partial f}{\partial \theta_{j}}+\frac{1}{2}tr\left[C^{-1}\left(\theta \right)\frac{\partial C^{-1}\left(\theta \right)}{\partial \theta_{i}}C^{-1}\left(\theta \right)\frac{\partial C^{-1}\left(\theta \right)}{\partial \theta_{i}}\right]$$ (30)

where **f** is the mean response of the population, and **C** the covariance of the population response. In our case, ***θ*** = [*ϕ* *ψ*]^*T*^, so the Fisher information is a 2-by-2 matrix.

Consider the case that the memory only contains a single item, with *β* = 1. Then

$$y_{N}\;|\;\phi ,\psi \;∼\;N\left(\mu \left(\phi ,\psi \right)\;,\;{\tilde{\Sigma }}_{N}\right)$$ (31)

where we assume ${\tilde{\Sigma }}_{N}=\sigma_{x}^{2}I$. Since the covariance ${\tilde{\Sigma }}_{N}$ does not depend on *θ*, the trace term in the Fisher information is 0.

The FI about the angle is given by

$${\left[I_{F}\left(\theta \right)\right]}_{\phi \phi }={\frac{\partial \mu }{\partial \phi }}^{T}\frac{1}{\sigma^{2}}I\;\frac{\partial \mu }{\partial \phi }$$ (32)

$${\left[\frac{\partial \mu }{\partial \phi }\right]}_{i}=-\frac{\tau_{1}\sin\left(\phi -\theta_{i}\right)}{4\pi^{2}I_{0}\left(\tau_{1}\right)I_{0}\left(\tau_{2}\right)}\exp\left[\tau_{1}\cos\left(\phi -\theta_{i}\right)+\tau_{2}\cos\left(\psi -\gamma_{i}\right)\right]$$ (33)

$$\Rightarrow {\left[I_{F}\right]}_{\phi \phi }=\frac{\tau_{1}^{2}}{\sigma^{2}16\pi^{4}I_{0}{\left(\tau_{1}\right)}^{2}I_{0}{\left(\tau_{2}\right)}^{2}}\overset{M}{\underset{i=1}{\sum }}\sin^{2}\left(\phi -\theta_{i}\right)\exp\left[2\tau_{1}\cos\left(\phi -\theta_{i}\right)+2\tau_{2}\cos\left(\psi -\gamma_{i}\right)\right]$$ (34)

The other components of the Fisher information matrix can be derived similarly.

By taking a large population limit in which preferred values have density *ρ*, we obtain a closed-form approximation to the Fisher information (see Section, 1 in S1 Text for the complete derivation):

$$\underset{M\to \infty }{\lim}{\left[I_{F1}\right]}_{\phi \phi }\approx \frac{\tau_{1}^{2}\rho }{\sigma^{2}8\pi^{2}I_{0}{\left(\tau_{1}\right)}^{2}I_{0}{\left(\tau_{2}\right)}^{2}}I_{0}\left(2\tau_{2}\right)\left(I_{0}\left(2\tau_{1}\right)-I_{2}\left(2\tau_{1}\right)\right)$$ (35)

### Parameter optimization

We perform a grid search over several population code parameters to provide a qualitative fit to human experiments. For the mixed population code, we varied *σ*_*x*_ and the *ratio* of conjunctivity, as *β*, *σ*_*y*_ were kept fixed. For the hierarchical code, we set *p* = 1, *λ* = 1 and Θ = 1 and varied *σ*_*x*_ and the *ratio* of conjunctivity (defined as $\frac{M_{2}}{M_{1}+M_{2}}$, where *M*_1_ (respectively *M*_2_) is the size of the layer one subpopulation (respectively layer two)). A full fit, which is the subject of future work, would require at least the consideration of heterogeneous and multi-scale population representations.

## Supporting Information

S1 Text Supplementary Material. Additional derivations and results omitted from main manuscript. Derivations include the computation of the large population limit for Fisher information and the relation between the memory fidelity and the Fisher information. We report the stimuli separation analysis for the hierarchical code, analogous to the analysis of Fig. 16 in the manuscript. Following the comments of a reviewer, we studied the relationship between the conjunctivity ratio and the population size in a mixed population code, as our parametrisation creates a dependence between them. Finally, we show how increasing the number of features affects the ratio of conjunctivity for a fixed population size.
(PDF)
