# Supplementary material for: A Probabilistic Palimpsest Model of Visual Short-term Memory
Source: PLoS Comput Biol. 2015 Jan 22;11(1):e1004003. doi: 10.1371/journal.pcbi.1004003 (PMC4303260; doi:10.1371/journal.pcbi.1004003)
Supplement: S1 Text — Additional derivations and results omitted from main manuscript. Derivations include the computation of the large population limit for Fisher information and the relation between the memory fidelity and the Fisher information. We report the stimuli separation analysis for the hierarchical code, analogous to the analysis of Fig. 16 in the manuscript. Following the comments of a reviewer, we studied the relationship between the conjunctivity ratio and the population size in a mixed population code, as our parametrisation creates a dependence between them. Finally, we show how increasing the number of features affects the ratio of conjunctivity for a fixed population size. (PDF) [file pcbi.1004003.s001.pdf]

Supplementary Text S1

1 Derivation of large population limit for Fisher information

Starting from Equation (8) in the main text, assuming uniform coverage with density ρ for the population code.

$$
\begin{aligned}
[\mathbf{I_F}]_{\phi\phi} &= \frac{\tau_1^2}{\sigma^2 16\pi^4 I_0(\tau_1)^2 I_0(\tau_2)^2} \sum_{i=1}^{M} \sin^2(\phi-\theta_i) \exp\left[2\tau_1\cos(\phi-\theta_i) + 2\tau_2\cos(\psi-\gamma_i)\right] \\
&\approx \frac{\tau_1^2\rho}{\sigma^2 16\pi^4 I_0(\tau_1)^2 I_0(\tau_2)^2} \int_0^{2\pi}\int_0^{2\pi} d\gamma d\theta \sin^2(\phi-\gamma)\exp\left[2\tau_1\cos(\phi-\gamma)\right. \\
&\quad \left. + 2\tau_2\cos(\psi-\theta)\right] \\
&= \frac{\tau_1^2\rho}{\sigma^2 16\pi^4 I_0(\tau_1)^2 I_0(\tau_2)^2} \int_0^{2\pi} d\gamma \sin^2(\phi-\gamma)\exp\left(2\tau_1\cos(\phi-\gamma)\right) \\
&\quad \cdot \int_0^{2\pi} \exp\left(2\tau_2\cos(\psi-\theta)\right) d\theta \\
&= \frac{\tau_1^2\rho I_0(2\tau_2)}{\sigma^2 8\pi^3 I_0(\tau_1)^2 I_0(\tau_2)^2} \left(2\pi I_0(2\tau_1) - \int_0^{2\pi} d\gamma \cos^2(\phi-\gamma)\exp\left(2\tau_1\cos(\phi-\gamma)\right)\right)
\end{aligned}
$$

With:

$$
\begin{aligned}
&\int_0^{2\pi} d\gamma \cos^2(\phi-\gamma)\exp\left(2\tau_1\cos(\phi-\gamma)\right) = \int_0^{2\pi}\cos^2(v)\exp(2\tau_1\cos v)dv \\
&\text{Introduce } \gamma, \text{and let } \frac{d^2}{d\gamma^2}\exp(2\gamma\tau_1\cos v) = 4\tau_1^2\cos^2 v\exp(2\gamma\tau_1\cos v) \\
&\Rightarrow \int_0^{2\pi}\cos^2(v)\exp(2\tau_1\cos v)dv = \frac{1}{4\tau_1^2}\int_0^{2\pi}\frac{d^2}{d\gamma^2}\exp(2\gamma\tau_1\cos v)dv\Big|_{\gamma=1} \\
&= \frac{1}{4\tau_1^2}\frac{d^2}{d\gamma^2}\int_0^{2\pi}\exp(2\gamma\tau_1\cos v)dv = \frac{\pi}{2\tau_1^2}\frac{d^2}{d\gamma^2}I_0(2\gamma\tau_1) \\
&= \frac{\pi}{2\tau_1^2}\frac{4\tau_1^2}{2}\left(I_0(2\gamma\tau_1)+I_2(2\gamma\tau_1)\right)\Big|_{\gamma=1} = \pi\left(I_0(2\tau_1)+I_2(2\tau_1)\right)
\end{aligned} \tag{1}
$$

Hence obtaining

$$
\lim_{M\to\infty}[\mathbf{I_F}_1]_{\phi\phi} \approx \frac{\tau_1^2\rho}{\sigma^2 8\pi^2 I_0(\tau_1)^2 I_0(\tau_2)^2} I_0(2\tau_2)\left(I_0(2\tau_1)-I_2(2\tau_1)\right) \tag{2}
$$

2 Relation between memory fidelity and Fisher information

In the "Fisher information analysis" main text section, we mentioned that the memory fidelity is approximately half of the Fisher information, which is the expected curvature of the log-posterior.

Assume that we store an item with a single feature θ in a memory comprising M units whose activities are denoted collectively by $\mathbf{y}$. Storage is noisy. We perform recall by constructing and then sampling from the posterior distribution $p(\theta \mid \mathbf{y})$. The mode of this distribution, $t(\mathbf{y})$ is a function of $\mathbf{y}$, and in the cases of interest, will often be an an unbiased estimator of θ, saturating the Cramer-Rao bound, so that

$$E\left[t(\mathbf{y}) \mid \theta\right] = \theta \tag{3}$$

$$\text{Var}\left[t(\mathbf{y}) \mid \theta\right] = \frac{1}{FI(\theta)} \tag{4}$$

If the prior over θ is flat, then the posterior distribution, given $\mathbf{y}$, will, in these circumstances, be well approximated by a Gaussian distribution with mean $t(\mathbf{y})$ and the same variance $\frac{1}{FI(\theta)}$. If we take a sample $\hat{\theta}$ from this, then we have

$$E\left[\hat{\theta}|\theta\right] = E\left[E\left[\hat{\theta}|\mathbf{y}\right]|\theta\right] = E\left[t(\mathbf{y})|\theta\right] = \theta \quad \text{by iterated expectations} \tag{5}$$

$$E\left[(\hat{\theta}-\theta)^2|\theta\right] = E\left[(\hat{\theta}-t(\mathbf{y}))^2 + (t(\mathbf{y})-\theta)^2|\theta\right] \quad \text{since } \hat{\theta} \text{ is sampled independently of } \mathbf{y} \tag{6}$$

$$= E\left[Var\left[\hat{\theta}|\mathbf{y}\right]|\theta\right] + Var\left[t(\mathbf{y})|\theta\right] \tag{7}$$

$$= \frac{1}{FI(\theta)} + \frac{1}{FI(\theta)} = \frac{2}{FI(\theta)} \tag{8}$$

as claimed.

3 Stimuli separation analysis for Hierarchical population code

We perform a similar analysis as for the mixed population code in "The canary" main text section, in which the patterns of error were used to make inferences about the underlying size of the receptive fields of the units in the population (under various assumptions). See Figure 1 in Text S1 for the results. For most conjunctivity ratios, the separation between target and non-target recall occurs for a minimum distance of 0.2. However, the performance saturates at a level that depends on the conjunctivity. Hence for a given hierarchical conjunctivity, moving the stimuli further apart does not improve recall arbitrarily. This can be interpreted as being caused by the non-locality of the conjunctive information in the hierarchical code. Increasing the distance between the stimuli does not change the probability that it falls into a conjunctive receptive field.

4 Relation between conjunctivity ratio and population size

A reviewer noted that our parameterisation of the mixed population code creates a dependence between the population size M and the ratio of conjunctivity required to achieve a particular precision. For instance, the fidelity of feature-selective neurons scales as M, while the precision of conjunctive neurons scales as M^2 (for two features). Hence from a purely theoretical point of view, the ratio of conjunctivity should scale with M.

We tested this hypothesis by looking at storage and recall of just a single item in a mixed population code, modifying the population size M and the ratio of conjunctivity. The results are shown in Figure 2

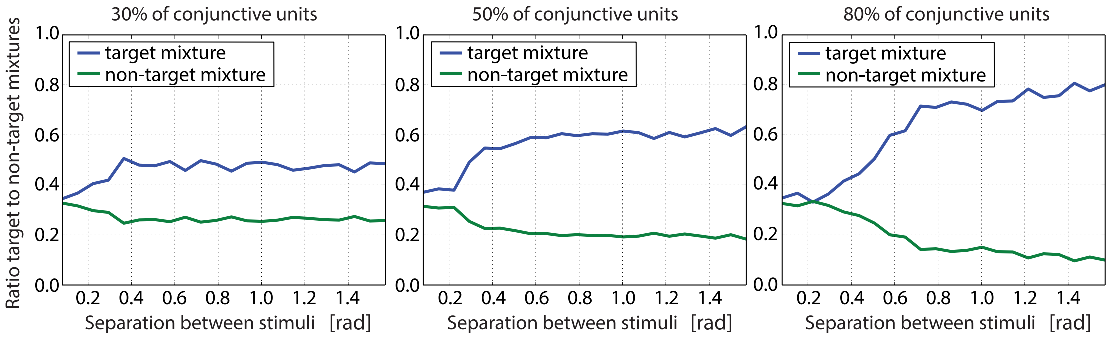

Figure 1. Patterns of errors as a function of stimulus separation for different proportions of conjunctive units in the hierarchical population code.
This figure is a version of Figure 16 in the main text, but for a hierarchical rather than mixed population code. It shows the ratios between the target mixture proportion and the sum of the target and non-target mixture proportions (in blue), or the same for a non-target mixture proportion (in green) as a function of the separation in radians between stimuli in the diagonal pattern, for three hierarchical population codes, with 30%, 50% and 80% conjunctive units. See Supplementary information 3 for analysis.

in Text S1 in terms of the precision (quantified by the memory error since, as explained in the paper, the circular standard deviation is too sensitive to outliers). This surface is based on an interpolation between a set of discrete values of M that fit with the homogeneity of the code.

The ratio necessary to achieve a given $\kappa = \hat{\kappa} = 580$ did indeed scale with M, at least for most values. Increasing the population size further led to precisions that were too high, even when increasing the conjunctivity to its maximum value (hence our target $\hat{\kappa}$ is not reachable).

Note that we kept M fixed almost throughout the paper, and also that it may not be that human performance is best fit by using the M that achieves the best precision. Indeed, in our ongoing work on this, it currently seems as if suboptimality holds.

We also reconfirmed the good fit of the Fisher information to (twice) the memory fidelity κ. Figure 3 in Text S1 shows the ratio of the Fisher information to the value that corresponds to the same $\hat{\kappa}$ shown in Figure 2 in Text S1; it follows exactly the same trend. Unfortunately, as the Fisher information is a complex function of the ratio of conjunctivity in the mixed population code, its evolution with M does not follow any simple relationship.

5 Relation between ratio of conjunctivity when increasing number of features

For similar reasons, the required fraction of conjunctive units for a given κ is also linked to the number R of feature dimensions. As noted in the main text, although N^R conjunctive units are required to cover the space evenly, only $N \cdot R$ feature units are required. This comes at the cost of misbinding. Hence it can be expected than the ratio of conjunctive units that keeps κ fixed should decrease as R increases.

Figure 4 in Text S1 shows the effect of varying R, keeping the population size M fixed (to $M = 356$ in this experiment), and adjusting the ratio of conjunctivity. We tried cueing all the features apart from

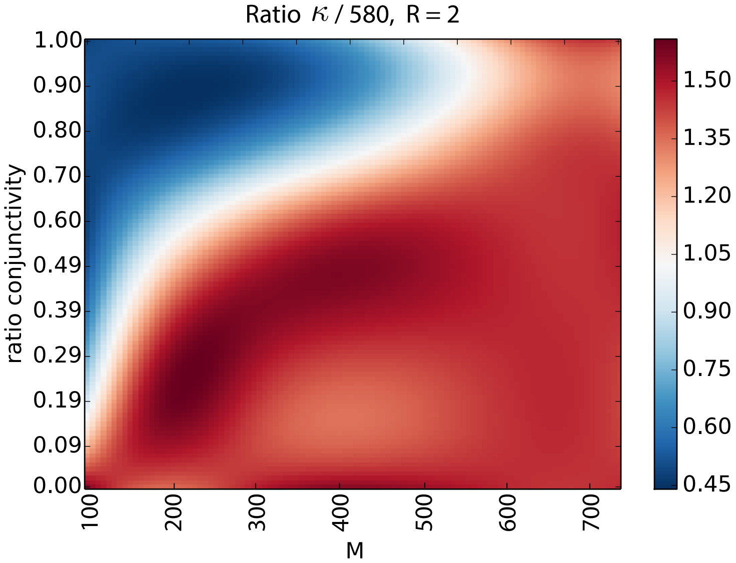

Figure 2. Precision as a function of M and the ratio of conjunctivity.
The plot shows the dependence of the precision (measured, for convenience, as the ratio $\kappa/\hat{\kappa}$ relative to a specific value $\hat{\kappa} = 580$) for a single item as a function of the size of the population code, M, and the ratio of conjunctivity. White corresponds to a ratio $\kappa/\hat{\kappa}$ of 1; we clamped the ratios between 0.5 and 1.5 for visualisation purposes (ratios smaller than 0.5 are visualized as $= 0.5$, ratios larger than 1.5 are visualized as $= 1.5$). This surface is an interpolation between the points that are possible (to maintain the homogeneity of the population representation). We see that the white equal-ratio ridge does not reach the top-right corner and instead seems to "reverse". This is due to the impossibility to match the specific value of $\hat{\kappa}$ for large population sizes.

the one to be recalled (hence simplifying the problem to be trivially similar to the situation with $R = 2$) and also tried to cue a single feature (doing Gibbs sampling on all the remaining ones). As no clear qualitative difference was found, except that the single cued feature case was (as expected) less efficient, we only report our results for the fully cued case.

We found that the overall precision achievable by the network decreases with R. Hence to achieve a given precision level, it makes it appear as a smaller ratio is required. However, this does not take misbinding errors into account.

To show how misbinding occurs, we also checked how the mixture proportion associated with on-target response changed. As shown in Figure 4 in Text S1 (bottom row), responses were on target for highly conjunctive populations, decreasing as the ratio of conjunctivity decreased. It can also be seen that increasing the number of features has an effect, requiring a greater degree of conjunctivity as the number of features increases. There does not seem to be a simple relationship between the probability of correctly binding the features together and the number of features and conjunctivity.

We found it quite complicated to construct a single metric that would appropriately combine the statistics shown in the top and bottom of Figure 4 in Text S1. The precision of the errors, however promising, is too sensitive to outliers, as discussed in the main text. Hence we keep the two metrics separate for this current work.

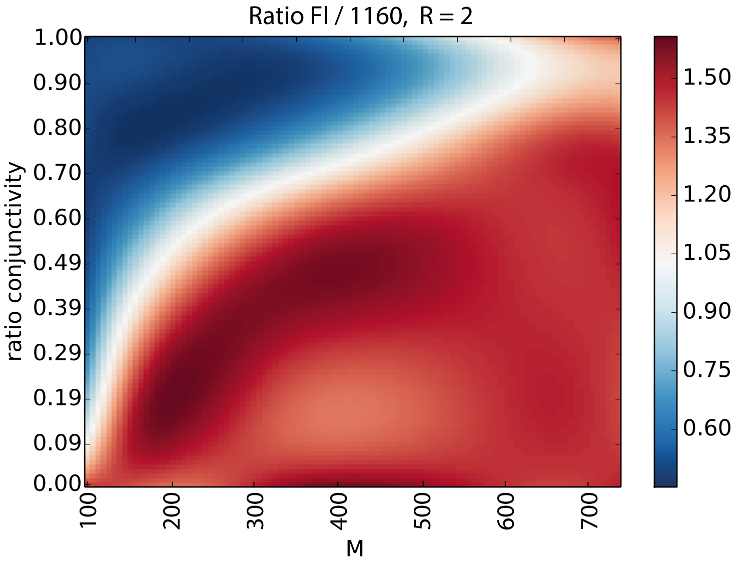

Figure 3. Fisher information as a predictor of memory fidelity
The plot shows the Fisher information calculated using exactly the same parameters and cases as in Figure 2 in Text S1. The two figures match closely. Unfortunately, the Fisher information is a complex function of the ratio of conjunctivity in the mixed population code, and it does not follow an obvious closed-form relationship with M.

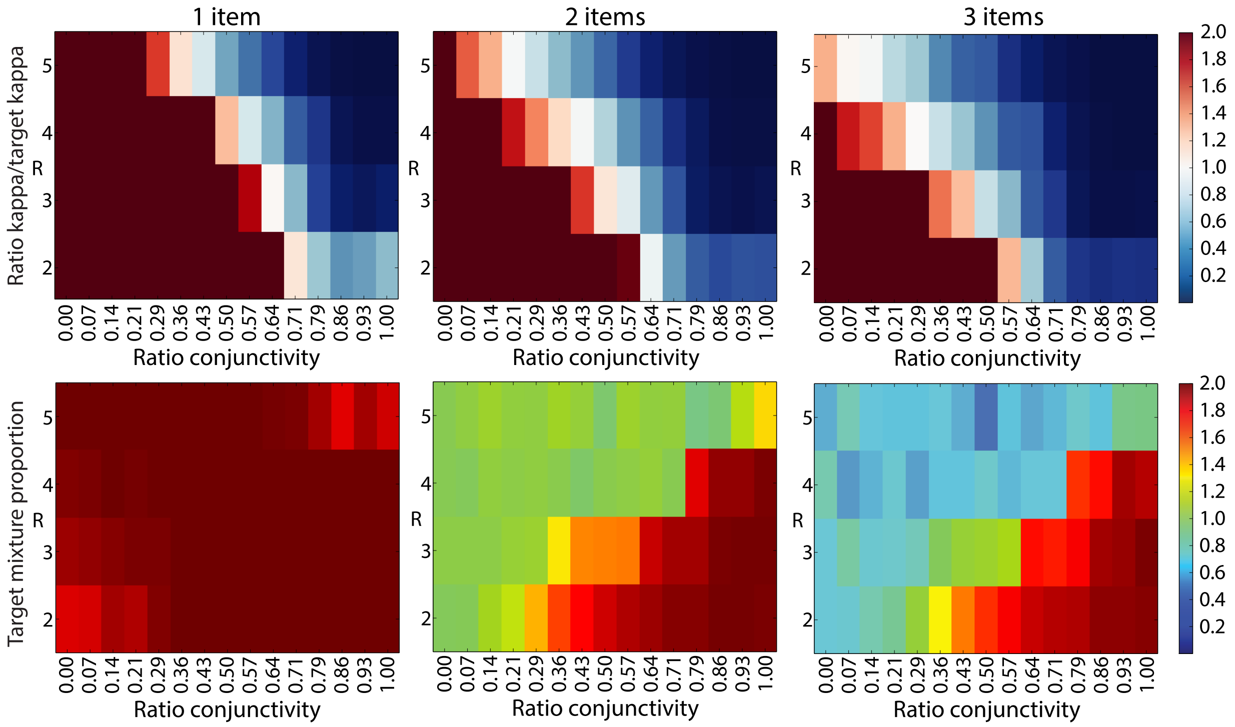

Figure 4. Effect of $R > 2$ on the precision.
We simulated the model for 1, 2 and 3 items, and for various numbers of features R and ratios of conjunctivity, for a fixed size of network ($M = 356$; for illustration). **Top row**: Ratio of the obtained memory precision/fidelity κ to an arbitrary target precision of $\hat{\kappa} = 580$. As the overall precision κ decreases with R, the ratio required to achieve a give precision also decreases with R. **Bottom row**: Mixture proportion associated with responses to the target. This decreases dramatically as the number of items or features increases.
